# Impact of electrolyte impurities and SEI composition on battery safety†

**DOI:** 10.1039/d3sc04186g

**Published:** 2023-11-03

**Authors:** Florian Baakes, Daniel Witt, Ulrike Krewer

**Affiliations:** a Institute for Applied Materials – Electrochemical Technologies, Karlsruhe Institute of Technology Adenauerring 20b 76131 Karlsruhe Germany ulrike.krewer@kit.edu

## Abstract

Li-ion batteries have a potential risk of thermal runaway. Current safety evaluations in academia and industry rely on experiments or semi-empirical simulations. This limits the understanding of processes leading to or occurring during thermal runaway and how chemical species and impurities can impact them. The limited (quantitative) understanding in turn hinders a holistic safety assessment and optimisation of countermeasures through design or operation. The here presented thermal-runaway model contains a detailed degradation reaction network, which allows the impact of chemical species and impurities on thermal runaway to be studied. We set a particular focus on water impurities and solid-electrolyte interphase (SEI) properties, as both are known to impact life-time of batteries. SEI composition and thickness change during ageing, which is shown here to impact battery safety significantly. The model can reproduce reported experimental behaviour: aged cells are more safe, as they start self-heating, *i.e.* heat production without an external heat source, at 15–20 °C higher temperatures than fresh cells. Our model suggests a thick inorganic and thus less reactive SEI as the underlying cause. Furthermore, we could show that extensive electrode drying to remove water impurities before building battery cells will not significantly improve safety characteristics. In contrast, electrodes not subjected to any drying procedure cause an earlier start of the self-heating phase, *i.e.* have a higher risk of thermal runaway. These insights into the sensitivity to thermal runaway allow robust methods to be tailored for its prevention, from controlling battery and SEI properties during production to adjusting safety assessment for effects of ageing.

## Abstract 2

Our work highlights the impact of impurities and SEI properties on Li-ion battery safety. We could show that a thicker, inorganic SEI leads to a significant delay in thermal runaway, indicating a safer aged battery.

## Graphical abstract

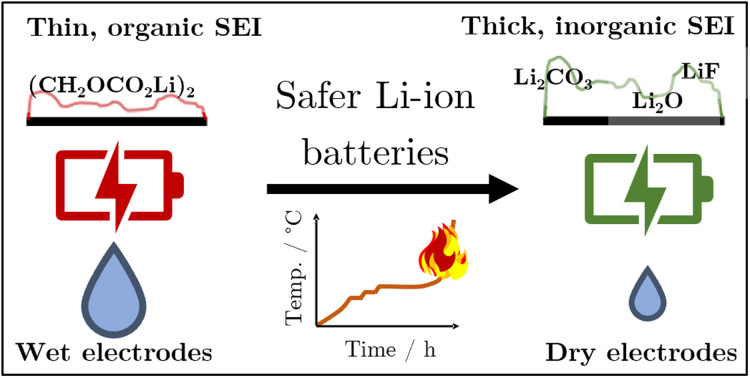

## 1 Introduction

The increase in electromobility demands cost-effective and safe Li-ion battery materials.^1^ With ever-increasing energy densities, safety aspects in Li-ion batteries receive more and more attention from researchers.^2^ The most critical safety issue with Li-ion batteries is thermal runaway. This phenomenon of uncontrolled temperature increase in the battery starts with slow self-heating, *i.e.* heating without external heat sources, and can end in explosions, fires, and cell temperatures above 800 °C.^3^

Especially the initial period of self-heating provides an opportunity to apply counteracting measures to prevent thermal runaway. Yet, designing effective countermeasures demands a thorough understanding of the processes triggering and occurring during this critical initial period. In a previous study, we could show that the self-heating phase is characterised by an intricate balance between endothermic and exothermic processes.^4^ The main exothermic reactions are related to the decomposition and reformation of the solid electrolyte interphase (SEI), a protective layer at the surface of carbon-based negative active material particles.^4,5^ Presently, not much is known about the effect of SEI properties and highly reactive impurities such as water on the self-heating behaviour of Li-ion batteries. This motivates us to conduct a detailed analysis in this publication.

Since its discovery in the late 1980s, the SEI is among the most investigated parts of Li-ion batteries.^6–8^ As a naturally formed surface layer on the carbon electrode, it originates from solvent and conductive salt reduction during the first charge–discharge cycle, *i.e.*, during the so-called cell formation process. After formation, the SEI serves as an electric isolator in the ion-conducting phase. Stabilising the SEI and preventing its continuous growth due to ongoing SEI reactions is crucial for reaching long battery life. Tremendous efforts have been undertaken to learn more about its composition, structure, and effects on performance.^7–9^ In a recent study, Liu *et al.*^10^ combined electrochemical measurements, an electrochemical quartz crystal microbalance, atomic force microscopy, X-ray photoelectron spectroscopy, and online electrochemical mass spectrometry to estimate the composition and thickness of the SEI after formation. With this combined approach, they were the first to show that the initially formed organic SEI component lithium ethylene dicarbonate (LEDC) is partially oxidised to form Li_2_O and several gases. However, the influence on battery performance was not investigated in their study. Son *et al.*^11^ compared the influence of the SEI-forming additives fluoroethylene carbonate (FEC) and vinylene carbonate (VC) on battery performance. They found that FEC could enable better fast-charging capabilities and long-term capacity retention. This is assumed to be connected to a higher resistance for the cell with VC, which is caused by a more polymeric SEI stemming from VC as an additive. The higher resistances on the electrode surface lead to higher overpotentials at elevated currents, eventually resulting in lithium plating during charging. As the SEI is a nanometer-thin surface layer, experimental investigations are very complex,^12^ and quantitative analysis has not yet been possible. Thus, a plethora of simulation methods have been used to tackle this problem.^13^

In a simulation study using a kinetic Monte-Carlo (kMC) approach connected to a Newman-type battery cell model, Röder *et al.*^14^ showed that higher currents during initial SEI formation may lead to a gradient in the SEI thickness along the electrode thickness. With a holistic model-based analysis of experimental electrochemical impedance spectroscopy data and C-rate tests, we could relate the ageing behaviour of Li-ion cells, among other factors, to an evolution of SEI thickness and interfacial properties.^15^ The model contained a Newman-type cell model extended with an SEI. In a recent study Esmaeilpour *et al.*^16^ used a kMC approach with over 50 000 simulation conditions to find that the most probable way of SEI formation is a sequence of formation, dissolution and finally, aggregation. Furthermore, approaches using kMC methods in combination with computational chemistry support long-time existing hypotheses about the composition and morphology of the SEI.^17–19^ These studies illustrate how simulations can be used as powerful tools to unravel, supplement or support existing theories on SEI growth and functionality.

It was already 20 years ago when the first experiments by Dahn *et al.* connected the self-heating behaviour of Li-ion cells to SEI decomposition.^5^ In experimental studies of aged cells, an increase in the self-heating temperature of about 15–20 °C had been suggested to stem from a thicker and more inorganic SEI.^20–23^ However, neither detailed experimental nor simulation-based studies have so far been performed on the influence of SEI composition or thickness on battery safety, especially during the self-heating phase. Thus, this publication aims to tackle this open challenge.

H_2_O is a known impurity during battery manufacturing.^24^ Hygroscopy of electrodes is the most often referenced origin of water in cells.^25,26^ Several studies showed the detrimental effect of water on battery performance.^26–28^ However, also positive impacts of H_2_O addition for Li-metal batteries have been reported.^29^ LiF is formed from the reaction of H_2_O with LiPF_6_, and it is a stable and highly conducting SEI component leading to better performance. In contrast, worse performance is usually connected to formation of the phosphorous decomposition products PF_5_ and POF_3_. These reactions are known to occur at room temperature and are inherently linked to battery ageing. Weber *et al.*^30^ investigated the ageing of an electrolyte mixture stored at 95 °C and identified 12 different organo-phosphoric decomposition products. They suggested that a detailed analysis of the formation of these products during ageing could be used to identify “ageing stages” for LiPF_6_-based electrolyte composition. Stich *et al.*^31^ investigated the kinetics of these decomposition reactions by purposely contaminating an electrolyte solution with 1000 ppm water and measuring the concentrations of H_2_O, HF and HPO_2_F_2_. They found that hydrolysis is not following a simple rate law and thus developed a kinetic model to describe their experiments. Huttner *et al.*^32^ studied the influence of different drying strategies for water removal on battery performance. They found that extensive drying can decrease performance. This is due to the extreme conditions the battery materials are subjected to during drying. In general, H_2_O contamination and follow–up reactions might not immediately influence battery performance, as Zheng *et al.* showed.^28^ They found that after 100 cycles, the capacity retention for batteries contaminated with H_2_O and without contamination was in the same range, with around 95% remaining capacity for water-free and 90% remaining capacity for water-containing batteries. However, after 300 cycles, increased H_2_O content drastically reduced capacity retention. The results show 90% remaining capacity for water-free batteries and 55% for water-contaminated ones. The water-containing battery had 14 mg water added to an 18650 battery. Despite these efforts to qualitatively and quantitatively correlate H_2_O contamination and battery performance, we have not found any reports in the literature on tests to investigate the effect of water on battery safety, especially during the crucial self-heating phase. This contribution will shed light on the extent to which H_2_O contamination influences battery safety.

To address both the sensitivity of water impurities and of SEI composition to thermal self-heating and thermal runaway, we use a component-based Li-ion battery degradation model. This approach allows us to assess the impact of each participating chemical species on the progression towards thermal runaway at open circuit potential. Our comprehensive model encompasses 12 decomposition reactions and 20 participating species, which will be parameterised through two separate experiments. First, for the conductive salt decomposition, the experiments conducted by Stich *et al.*^31^ will be used. The complete set of reactions is then parameterised against an accelerated rate calorimetry (ARC) measurement.^33^ The thus parameterised model is then used to conduct in-depth case studies on the effect of LEDC content in the SEI, the SEI thickness, and the H_2_O contamination on self-heating and thermal runaway. Eventually, we present a broader parameter study that combines all three effects, examining potential interdependencies between them and illustrating dominant processes and properties.

## 2 Methods and study cases

This section outlines the underlying reaction network. Furthermore, it provides a detailed description of the model with its assumptions, and the experiments employed for model parameterisation and validation. Eventually, the case study as well as the initial conditions of the reference case are introduced.

### 2.1 Reaction network

The reaction network of SEI and electrolyte degradation forms the core of this comprehensive case study as it allows for the traceback of thermal behaviour to single reactions, reaction interactions and species. The subsequent section provides a thorough overview of the postulated reactions, citing the relevant literature and discussing explicit and implicit interactions. Table 1 summarises the reactions, including the abbreviations used in this work, their reaction enthalpies and the prospective temperature range of a notable start of each reaction.

**Table 1:** Degradation reactions, their reaction enthalpy (blue = endothermic, red = exothermic) and the temperature range at which they are observed in differential scanning calorimetry, accelerated rate calorimetry or electrochemical measurements. Products that are also reactants are highlighted in bold^a^

Name	Abbr.	Equation	Δ_r_***H̲***/kJ mol^−1^	*T*_Start_/°C
Conductive salt decomposition^29^	CSD	LiPF_6_ ⇌ LiF + **PF**_**5**_	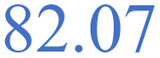	25*/60–80
PF_5_ decomposition^29^	PFD	PF_5_ + H_2_O ⇌ 2**HF** + **POF**_**3**_	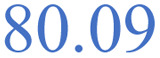	25*/60–80
POF_3_ decomposition^29^	POFD	POF_3_ + H_2_O → **HF** + HPO_2_F_2_	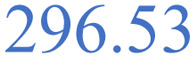	25*/60–80
Organic SEI production^30,31^	OSP	2LiC_6_ + 2C_3_H_4_O_3_ (EC) → (**CH**_**2**_**OCO**_**2**_**Li**)_**2**_ + C_2_H_4_ + 2C_6_	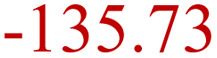	25**/80–120
Inorganic SEI production^30,31^	ISP	2LiC_6_ + C_3_H_4_O_3_ (EC) → **Li**_**2**_**CO**_**3**_ + C_2_H_4_ + 2C_6_	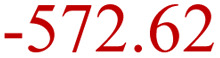	25**/80–120
LiOH production^20^	LSP	LiC_6_ + H_2_O → **LiOH** + 0.5H_2_ + C_6_	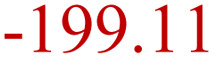	25**/80–120
Organic SEI decomposition^5,31^	OSD	(CH_2_OCO_2_Li)_2_ → **Li**_**2**_**CO**_**3**_ + C_2_H_4_ + CO_2_ + 0.5**O**_**2**_	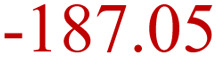	60–120
Inorganic SEI decomposition^32^	ISD	Li_2_CO_3_ + 2HF → 2LiF + **H**_**2**_**O** + CO_2_	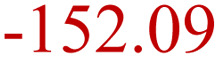	25
LiOH decomposition^33^	LSD	LiC_6_ + LiOH → Li_2_O + 0.5H_2_ + C_6_	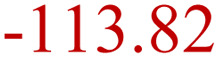	100–120
Cathode decomposition^34^	CD	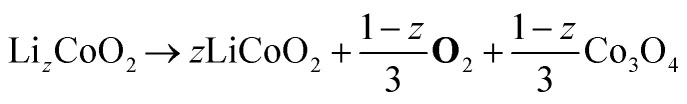	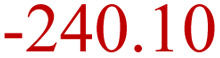	150–220
EMC combustion^34^	EMCD	3.5O_2_ + C_4_H_8_O_3_ (EMC) → 4CO_2_ + 4**H**_**2**_**O**	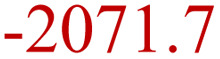	180–350
EC combustion^34^	ECD	2.5O_2_ + C_3_H_4_O_3_ (EC) → 3CO_2_ + 2**H**_**2**_**O**	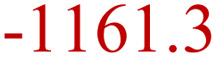	180–350

a * slow process during cell formation; ** process considered indirectly.

Considering thermal battery decomposition, the first reactions that occur during heating of the cells at notable rates are the conductive salt decomposition (CSD) and the subsequent decomposition to POF_3_ (PFD) and HPO_2_F_2_ (POFD). They are reported to occur at elevated temperatures around 60 °C to 80 °C. They also happen at room temperature, but only slowly and in the range of days, much longer than during thermal abuse, where reactions occur within hours or minutes. Following the publication of Stich *et al.*,^31^ we consider the decomposition of LiPF_6_ to PF_5_ and LiF (CSD) and the subsequent decomposition of PF_5_ with H_2_O to POF_3_ and HF (PFD) as equilibrium reactions. In their work,^31^ PF_5_ directly reacts with two H_2_O to form three HF and HPO_2_F_2_. However, in a study by Solchenbach *et al.*,^34^ POF_3_, an intermediate, was detected by online electrochemical mass spectrometry. This detection implies that the lifespan of POF_3_ is sufficiently long to be considered significant and cannot be disregarded in our analysis. Thus, we consider the first decomposition of PF_5_ with H_2_O to form POF_3_ and HF (PFD) and the subsequent decomposition of POF_3_ with H_2_O to form HF and HPO_2_F_2_ (POFD) as individual reactions.

The decomposition of LiPF_6_ and the subsequent reactions of its decomposition products PF_5_ and POF_3_ initiate the release of HF, which causes the breakdown of Li_2_CO_3_ by reacting with HF to form LiF, H_2_O, and CO_2_ (ISD). Freiberg *et al.*^35^ reported that Li_2_CO_3_ coated on a carbon electrode reacted immediately with protons, and thus HF, to release CO_2_. From this, we conclude that the decomposition reaction of Li_2_CO_3_ with HF or H^+^ is not rate-limiting and that it will happen rapidly as soon as LiPF_6_ is decomposed and releases HF.

The decomposition of the organic SEI component LEDC (OSD) is reported to start somewhere in a wide temperature range from 60 °C to 120 °C.^5^ It is responsible for the transition into the self-heating phase of an ARC measurement.^5,36^

The decomposition of LiOH with lithium metal or Li intercalated in graphite to form H_2_ and Li_2_O (LSD) is frequently reported in the literature.^25^ However, we could not find any information on the temperature range for this reaction. Thus, we depend on a study of the decomposition of LiOH with LiH into Li_2_O and H_2,_ which reported a decomposition temperature around 120 °C.^37^

The decomposition of SEI compounds, including Li_2_CO_3_, LEDC, and LiOH, eventually exposes the bare electrode surface. Given the solvent's instability against the anode's low potentials, subsequent SEI formation reactions occur at the electrode. These SEI-producing reactions (OSP, ISP, and LSP) are direct results of the preceding decomposition and are linked to the battery's self-heating.^31^

All reactions so far occur already below 130 °C and involve species in the anode or electrolyte. At significantly higher temperatures above 150 °C, self-heating is strongly accelerated by the decomposition of the cathode active material LiCoO_2_ (CD). The released O_2_, in turn, leads to the highly exothermic combustion of solvent molecules (ECD and EMCD),^36^ further accelerating thermal self-heating.

**Fig. 1:**
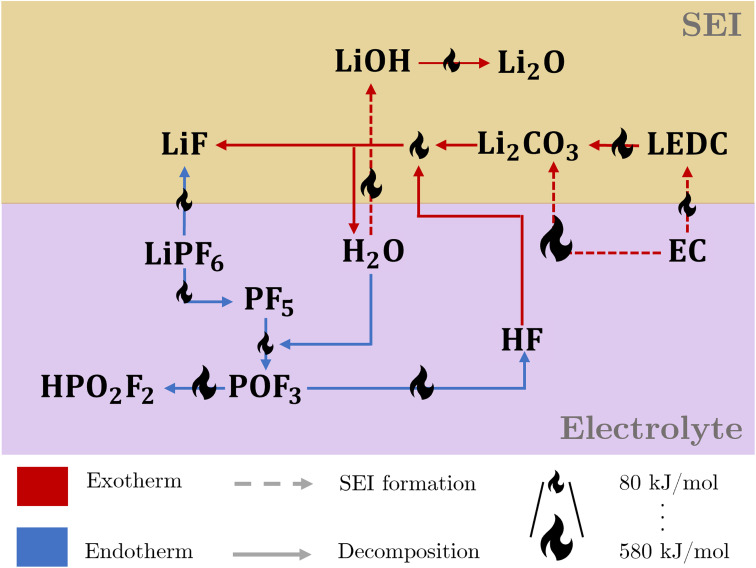
Reaction network for thermal composition of the SEI and electrolyte, covering the interaction between salt, solvent, and SEI. Red and blue arrows indicate exo- and endothermic reactions, respectively. The size of the flame icon on the arrows scales with the corresponding amount of consumed or released reaction heat.

Fig. 1 illustrates the intricate interaction of the thermal degradation processes at the electrolyte and the SEI. The reactions are strongly connected *via* the participating species, leading to a complex interconnected reaction network, including a reaction cycle involving HF and H_2_O.

### 2.2 Mathematical model

This section presents the modelling approach, the modelled system and the underlying assumptions. Our previous publication^4^ provides a more detailed description of the basic model.

#### 2.2.1 Model overview

The modelled thermal runaway scenario is that of a Li-ion battery subjected to an ARC test: here, the battery is heated step-wise, and if the battery is not heating up on its own during a resting phase without heating due to degradation reactions (self-heating), a further heating step is conducted. Temperature evolution is recorded. For more details, see the Parameterisation section. The reference experiment^33^ was conducted at open circuit voltage OCV = 4.15 V, corresponding to a state-of-charge of the battery of 100%. The battery was closed, *i.e.* no reactants could enter or leave the cell. Thus, the component balance in our model, eqn (1) in Table 2, accounts just for the reactions in Table 1, with no transport or further reactions with cell-external components. Reaction kinetics are based on an Arrhenius approach for almost all reactions, see eqn (2). Exceptions are the SEI formation reactions, *i.e.*, OSP, ISP, and LSP, where the active material LiC_6_ reacts with electrolyte components. These reactions are known to slow down with increasing SEI thickness, as seen in eqn (3). Besides the classical Arrhenius type temperature dependence we therefore introduce a dependency of SEI forming reaction kinetics on the SEI thickness. This dependency should be understood as the capability of the SEI to form an electron-insulating layer. This capability is reduced by its decomposition. However, crack formation induced by particle swelling^38–40^ and SEI dissolution^41^ could also lead to new SEI components. As these effects are not directly modelled, they are implicitly included in this formulation. Furthermore, the kinetics of the conductive salt decomposition (CSD) includes *α*, which is the dissociation constant of LiPF_6_. Additionally CSD and the subsequent decomposition of PF_5_ (PFD) are modelled as reversible reactions, see eqn (4) and (5). For both, the rate constant for the backward reaction is calculated based on the equilibrium constant, which in turn is computed from the Gibbs free energy of the respective reaction as seen in eqn (7)–(9).^42^ For the simulation of ARC measurements, we follow ref. 43, and we set the threshold for the battery self-heating rate to 0.02 K min^−1^. Furthermore, adiabatic conditions are assumed for the self-heating phase. From this, the energy balance is computed based on eqn (10). For the sake of brevity, the following dependencies are only briefly summarised. The corresponding model equations can be found in the ESI.† Temperature changes during self-heating and thermal runaway are driven by the heat of the single reactions. According to Hess' law, molar heat of reaction is computed from the enthalpies of formation of participating species. The heat consumption or production rate originating from each reaction is then calculated as the product of the molar heat of the reaction and reaction rate. The heat capacity of the battery is calculated from the individual components. For the calculation of concentrations and partial pressures and the distinction between their influence on reactions, four reference phases are assumed: the anode including the SEI, the liquid electrolyte, the cathode, and a gas phase. Here, the volume refers to the volume of the anode + SEI, electrolyte or cathode, depending on the species for which the concentration is calculated. Gas/liquid phase equilibrium is assumed. Eventually, the solubility of each gaseous species in the electrolyte is calculated assuming they stay in the liquid phase until the maximal solubility is reached. Further model assumptions are summarised in the following:

**Table 2:** Model equations to simulate the ARC test. They have been adopted from Baakes *et al.*^4^*Ω* is denoted as the set of species (SP), reactions (RE), a subset of species *i* participating in reaction *j* (U), gaseous species (G), liquid species (L), and solid species (S)

Description	Equation	No.
Species balance	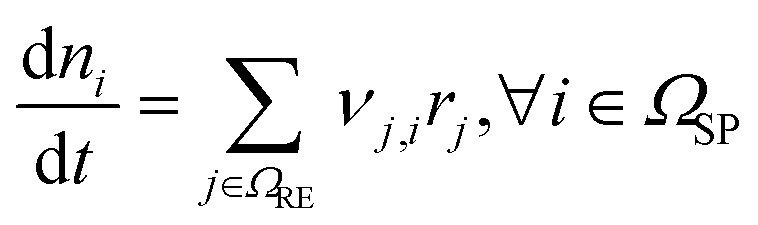	(1)
Reaction kinetics	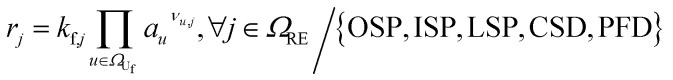	(2)
	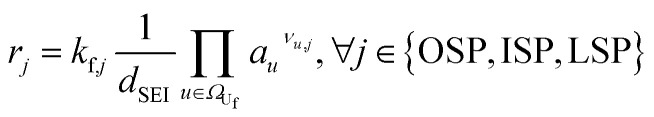	(3)
	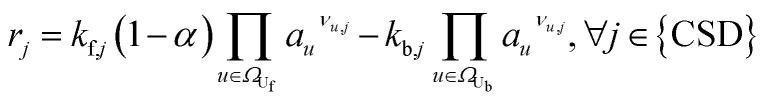	(4)
	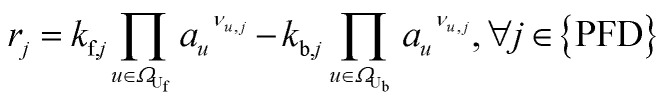	(5)
	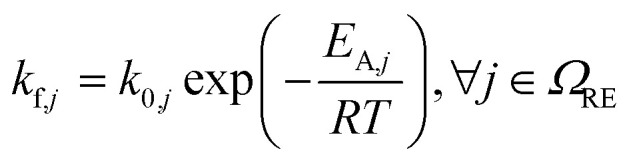	(6)
	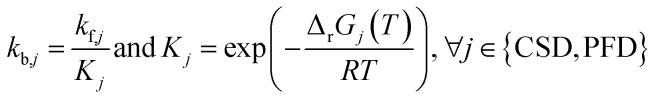	(7) and (8)
	Δ_r_*G*_*j*_(*T*) = Δ_r_*H*_*j*_(*T*) − *T*Δ_r_*S*_*j*_(*T*), ∀*j* ∈ {CSD, PFD}	(9)
Energy balance	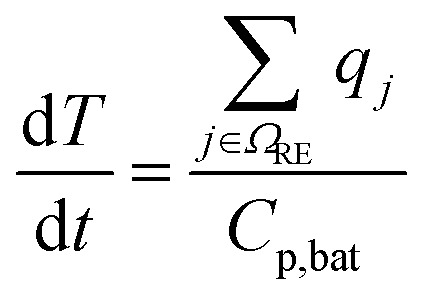	(10)

• All reactants are ideally mixed, so no spatial discretisation is applied. Since the time constant for diffusion and thermal conductivity is on the order of seconds and the ARC measurement takes hours, we deem the assumption of a perfectly mixed system a sound approach.

• A pressure increase due to gas evolution does not impact liquid-phase reactions.

• All reactions except for LiPF_6_ and PF_5_ decomposition are irreversible.

• SEI formation reactions are modelled as chemical reactions.

### 2.3 Experiments and parameterisation

This study aims to reveal the influence of H_2_O impurities and SEI composition on battery safety at high temperatures. Kinetic parameters, *k*_0_ and *E*_A_, are obtained from two distinct experimental studies: Stich *et al.*^31^ focused on the decomposition of LiPF_6_ with H_2_O as an impurity, providing an isolated study of LiPF_6_, PF_5_ and POF_3_ decomposition. Meanwhile, Maleki *et al.*^33^ performed an ARC measurement encompassing all concurrent reactions. In the following, the experiments themselves are described, as well as our parameterisation procedure.

Stich *et al.*^31^ used a 1 : 1 v:v EC:DEC 1 M LiPF_6_ electrolyte with an initial concentration of ≤ 15 ppm water and ≤ 50 ppm HF. They then added 1000 ppm of water. The concentrations of H_2_O, HF and HPO_2_F_2_ have been monitored for 15 days at room temperature by coulometric Karl Fischer titration, acid–base titration, and ion chromatography.

An ARC measurement by Maleki *et al.*^33^ is used to parameterise and study the transition from a safe battery at room temperature over its self-heating phase starting around 100 °C until the transition into rapid thermal runaway. The ARC measurements are conducted by inserting the battery into an oven-like apparatus. Following this, a linear temperature gradient is usually applied to reach a temperature where the first degradation reactions are expected to occur. In the experiments of Maleki *et al.*,^33^ this was 40 °C. Then, a heat–wait–seek (HWS) procedure is applied, where a 10 °C step-wise increase in temperature is conducted. Each step is followed by a period where the battery cell is equilibrating with the new temperature, and the temperature is monitored. No further heating is supplied when self-heating is detected during the seeking procedure. A detailed explanation of the underlying algorithm and the experiment used to parameterise our model is given in our previous publication.^4^ The cell used by Maleki *et al.*^33^ had a capacity of 550 mA h. The chemistry was reported to be a carbon-based anode, a LiCoO_2_ cathode, and polyvinylidene fluoride as the binder in both electrodes, and an ethylene carbonate (EC)/ethyl methyl carbonate (EMC) mixture with LiPF_6_ as the conductive salt. Since the original publication lacked some data crucial for simulation, the following assumptions have been made:

• The electrolyte consists of a 50 : 50 EC : EMC (v:v) mixture with 1200 mol m^−3^ LiPF_6_.

• The geometry of the cell is taken from a commercially available one that has a capacity and cell chemistry comparable to the cell used in the study by Maleki *et al.*^44^

• The electrode geometry is similar to other published electrodes of the same chemistry^45,46^

The kinetic parameters for the decomposition of LiPF_6_ and the subsequent reactions of PF_5_ and POF_3_, namely CSD, PFD, and POFD, have been parameterised such that two constraints are met. The first is that the experimental data^31^ of changes in the electrolyte at room temperature could be reproduced. The second is that the reactions notably accelerate within the reported temperature interval (see Table 1). For the decomposition of Li_2_CO_3_, the kinetic constants are set such that this reaction is not limiting.

For the other reactions, the kinetic parameters have been adjusted to meet two constraints: the first one is that the reactions occur within the reported temperature interval for each given reaction (Table 1). The second constraint is that the simulation can reproduce the experimental data from Maleki *et al.*^33^ Exceptions to this are the SEI forming reactions, OSP, ISP, and LSP. Given that they are, in nature, electrochemical reactions, they should notably occur even at room temperature with an unprotected electrode. Reported energy barriers for these reactions are very low, if not 0.^17,18,47^ The term introduced to account for the inhibition effect of the SEI (see eqn (3)) does not prevent notable reactions even at low temperatures with these low barriers. Therefore, the energy barriers are adjusted such that these reactions occur in a temperature range concurrently with the decomposition of the existing SEI.

The identified parameter values are given in the ESI.† Model equations and parameters were implemented in MATLAB, and the simulation was performed using the ode15s solver. All calculations have been performed with MATLAB Version 2022a,^48^ using an i7-9750H processor with 16 GB RAM. The average simulation time was six minutes.

### 2.4 Reference conditions and variation in water content and SEI properties

With their intricate internal decomposition mechanisms during formation, operation and thermal abuse, Li-ion batteries present a complexity that hinders the precise determination of the amount of species present at the beginning of a thermal decomposition study. Due to these restrictions, educated estimates for some components are necessary. For the sake of brevity, the procedures to arrive at the initial conditions will be shown in the ESI.† The values of interest are the conductive salt decomposition products, namely PF_5_, POF_3_, HPO_2_F_2_, HF, and H_2_O and the initial amount of the SEI components, Li_2_O, LEDC, LiOH, LiF, and Li_2_CO_3,_ as well as the initial SEI thickness, see Table 3.

**Table 3:** Changes in initial parameters for water content and SEI to study the effect of different manufacturing and ageing conditions: reference scenario, organic or inorganic SEI, thick or thin SEI, wet or dry electrode

Parameter	Scenario						
	Reference	Thick SEI	Thin SEI	Organic SEI	Inorganic SEI	Wet electrode	Dry electrode
*d*_SEI_/nm	50	**75**	**25**	50	50	50	50
*ε*_LEDC_/vol%	45	45	45	**90**	**0**	46	45
*ε*_Li_2_CO_3__/vol%	34	34	33	**8.4**	**39**	30	34
*ε*_LiF_/vol%	10.4	10.4	11.4	**1**	**30.4**	9	10.6
*ε*_Li_2_O_/vol%	10	10	10	**0**	**30**	10	10
*ε*_LiOH_/vol%	0.6	0.6	0.6	0.6	0.6	**5**	**0.4**
*C*_H_2_O_/ppm	260	260	260	260	260	**505**	**168**
*C*_HF_/ppm	0	0	0	0	0	**0**	**0**
*C*_PF_3__/ppm	992	992	992	992	992	**880**	**1017**
*C*_POF_3__/ppm	46	46	46	46	46	**95**	**31**
*C*_HPO_2_F_2__/ppm	1306	1306	1306	1306	1306	**5332**	**536**

H_2_O impurities, SEI compositions and SEI thickness can be influenced during manufacturing processes such as electrode drying or formation, as well as through battery ageing. At present, it is unfeasible to model and incorporate all these processes. Nevertheless, we implicitly investigate the effects and variability of different manufacturing conditions on battery behaviour during thermal abuse by designing a case study that uses various combinations of manufacturing and ageing inspired initial values for water impurity, SEI composition and thickness (Table 3). The variation in H_2_O content is based on a study of different drying procedures by Huttner *et al.*^32^ The H_2_O content will change due to different water contents in the electrodes following Huttner's results for the undried “wet”, the medium dried, and the highly dried electrodes. The different values in the anode of 2422 ppm, 286 ppm, and 214 ppm translate into 5 vol%, 0.6 vol% and 0.4 vol% of LiOH content within the SEI. The reported values of 2644 ppm, 500 ppm and 464 ppm in the separator and 313 ppm, 156 ppm, and 63 ppm for the cathode translate into an H_2_O increment in the electrolyte of 930 ppm, 334 ppm, and 172 ppm, respectively.

In the early 2000s it was revealed that LEDC is the primary decomposition product of EC.^49^ A recent study by Wang *et al.*,^50^ however, questions the stability of LEDC and proposed that lithium ethylene mono-carbonate (LEMC) is the stable alternative. Following this, Xie *et al.*^51^ investigated the formation pathways of LEMC, including the decomposition of LEDC and found that all kinetically favourable pathways need water as a reactant. It is apparent that there is still a lively discussion on the exact composition of the SEI. Thus, we will consider a wide range of differing compositions and assume LEDC as the major organic compound: here, 90 vol%, 45 vol% and 0 vol% LEDC are investigated. We did not choose the pure LEDC content since LiOH has to be part of the SEI to account for water impurities. Furthermore, the complete lack of Li_2_CO_3_ would lead to an accumulation of HF according to the above-described behaviour, which we deem very improbable. Thus, the remaining volume percentages are Li_2_CO_3_.

The SEI thickness has been varied between 25 nm, 50 nm, and 75 nm, which are within reported values for SEI thicknesses.^10,52,53^

All variations undergo the same formation and conditioning procedure described in the ESI.† From this, the initial values listed in Table 3 are calculated.

An extensive parameter variation, including all possible 27 variations (3 × 3 × 3), has been simulated to investigate the interdependence. The additional 18 initial values, apart from the 9 already listed in Table 3, can be found in the ESI.†

The initial and maximum temperatures are 25 °C and 220 °C, respectively. The pressure is constant at *p* = 101 325 Pa.

The initial conditions for all modelled species and their physical parameters and data on the electrode structure can be found in the ESI.†

## 3 Results and discussion

The upcoming section evaluates the propagation and characteristics of the thermal runaway during thermal abuse of the given Li-ion battery for the various manufacturing and ageing scenarios. First, sensitivities of ARC measurements to water content, SEI composition and SEI thickness are identified and quantified. This is followed by an in-depth analysis of this behaviour and the underlying causes based on the progression of reactions, their interplay, and their contribution to the temperature evolution during the abuse test. Particular focus is given to the transition from the heat–wait–seek to the exothermal self-heating mode, as this is the crucial point determining a battery's safety range. Then we elucidate the effects of different water contents and initial SEI properties on the state of the battery and occurring reactions and their consequences in an ARC measurement.

### 3.1 Impact of the SEI state and H_2_O on temperature evolution

**Fig. 2:**
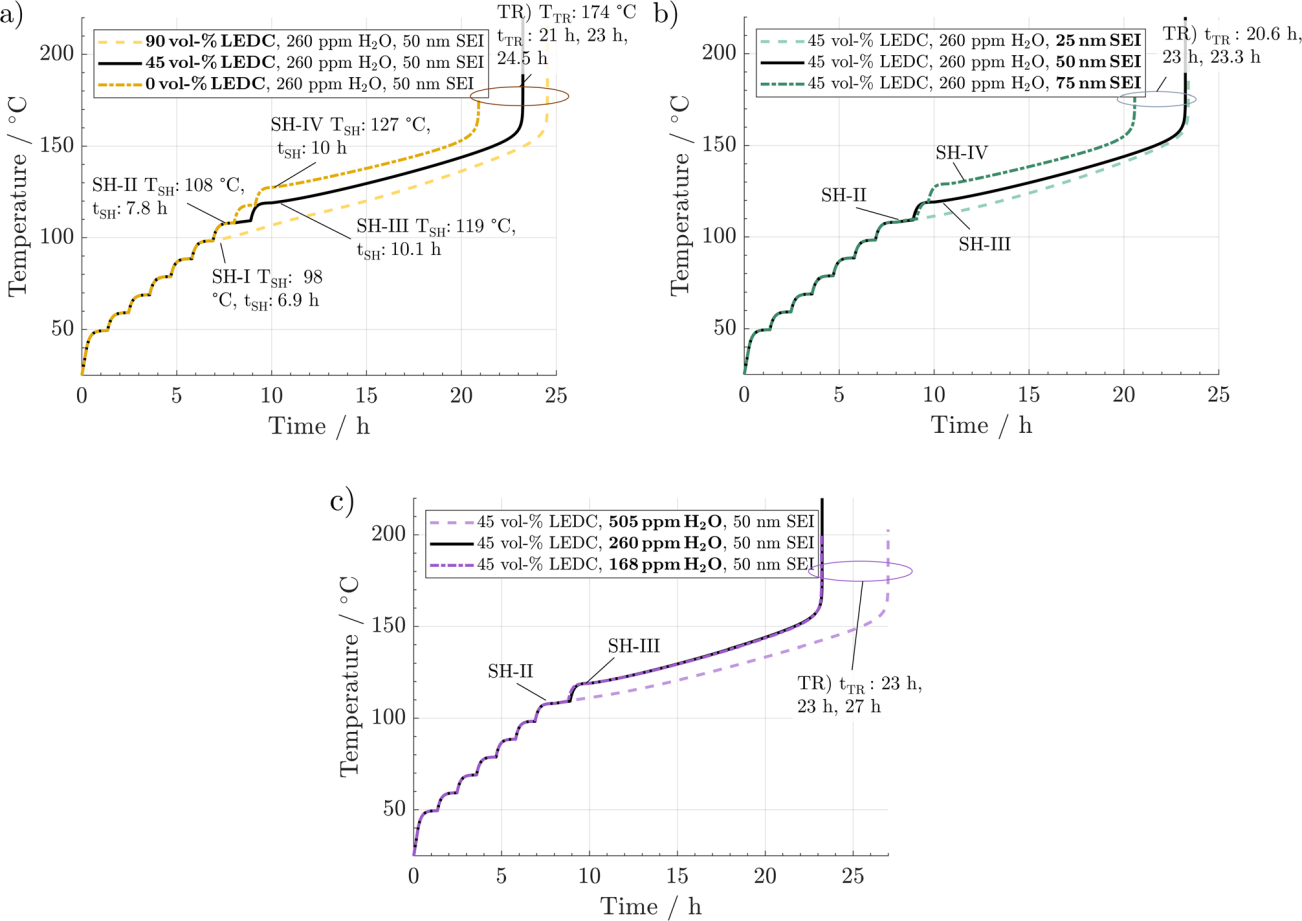
ARC simulations with variations of (a) SEI composition, (b) SEI thickness, and (c) water content originating from the electrodes. The solid black line refers to the reference case in all subfigures. The lighter dashed line refers to the scenario featuring the lower self-heating temperature *T*_SH_. The dash-dotted darker line indicates the scenario with the higher self-heating temperature. Characteristic self-heating temperatures, self-heating times, *t*_SH_, and runaway times, *t*_TR_, are indicated in the graphs.

In Fig. 2, the simulated temperature evolution is displayed for a variation in (a) SEI composition, (b) SEI thickness, and (c) H_2_O content. The preheating procedure (1 °C min^−1^) can be observed up to 40 °C. From then on, the ARC switches to the heat–wait–seek phase and performs 10 °C heating steps, including wait-and-seek periods. This is repeated until the first self-heating is detected. This happens for the reference scenario (black line) at 108 °C and 7.8 h, marked by SH-II. Then, the ARC switches to the self-heating mode and starts to follow the cell temperature. For the reference scenario, self-heating at this stage is not sustained, and after a period of 1 h at 110 °C, an additional heating step is performed. At 119 °C and 10.1 h, marked by SH-III, another self-heating of the cell leads to switching to the exothermic mode of the ARC again. The temperature monotonically increases throughout the subsequent 13 h until the thermal runaway is triggered at 174 °C and 23 h marked by TR.

Fig. 2a shows the effect of variations in SEI composition at 90 vol% LEDC (dashed line) and 0 vol% (dash-dotted line). No significant difference between the scenarios can be observed during the preheating phase and in the first six heating steps. At 98 °C and 6.9 h, marked by SH-I, significant self-heating is observed for the 90 vol% LEDC-case, which is 11 °C earlier compared to the reference case. Temperature increases monotonically without a further heating step, and the cell reaches the thermal runaway at 173 °C and 24.5 h. For the inorganic SEI without LEDC, one additional heating step compared to the reference case is needed, with self-heating starting only at 127 °C and 10 h. This makes a difference of +9 °C compared to the reference case. Yet, the cell goes faster into the thermal runaway. It can be concluded that SEI composition has a notable effect on thermal safety of a battery, with cells with more LEDC, *e.g.* due to less ageing, being more likely to enter a thermal event, leading finally to thermal runaway.

In Fig. 2b, the effect of SEI thickness on thermal runaway is presented. The behaviour during the preheating and first heating steps shows no deviation from that of the reference case. The thin SEI enters one heating step earlier into the self-heating phase than the reference case. Self-heating progresses and causes a rapid thermal runaway at 174 °C and 23.3 h. In contrast, the thick SEI case reaches the self-heating only after a further heating step, at around 127 °C and 10 h, and enters the final runaway phase earlier, at 174 °C and 20.6 h. According to this analysis, SEI thickness also significantly impacts battery safety, with a thicker SEI leading less quickly to self-heating. It should be noted that the results presented here are specific to the investigated system of a graphite anode combined with EC/EMC 1.2 M LiPF_6_ liquid electrolyte. For example, in the case of Li metal and all solid state batteries, a decrease in safety with increasing SEI thickness was found.^54,55^

Finally Fig. 2c shows the impact of H_2_O impurities. As in the variations before, no difference is observed until 108 °C and 7.8 h. Then, the cell with high H_2_O amounts enters one heating-step earlier into the sustained self-heating phase. The thermal runaway is reached at 174 °C and 27 h. In contrast, the curves for low and medium water amounts are identical. Thus, high amounts of H_2_O seem to be detrimental to thermal safety of Li-ion batteries.

**Fig. 3:**
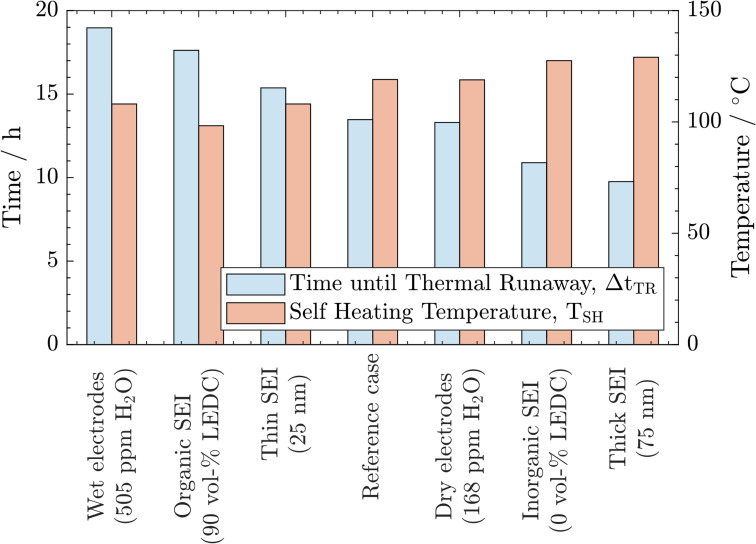
Comparison of self-heating temperature *T*_SH_ and time until the thermal runaway is reached after the first exothermic phase Δ*t*_TR_ as key indicators for all cases.

From the above-described results, we conclude that all three parameters significantly impact thermal safety. The most apparent difference between all cases is the change in self-heating temperature and the time from there until the thermal runaway is reached. For a better comparison, these key parameters are summarised in Fig. 3. Here, a clear trend can be observed. A lower self-heating temperature corresponds to a longer time until the cell reaches the thermal runaway eventually. These opposing trends of earlier self-heating but later thermal runaway pose a fundamental question as to how to produce inherently safer batteries: while higher self-heating temperatures can be interpreted as safer, a shorter time until reaching the thermal runaway, and with this, a virtually unstoppable thermal event could be considered unsafe. Manufacturers must perform a risk analysis to make a good trade-off for these safety-critical parameters. While discussing the case study, including 27 variations, in Section 3.3, we offer an alternative metric to include both characteristics in the safety assessment of Li-ion batteries. Note that the temperature for the start of the rapid thermal runaway is identical in all cases. Similarly, as reported in the literature, this is almost exclusively caused by the onset of cathode active material decomposition. In our study, the cathode material was not varied. Thus, this behaviour was expected.

In the following section, a deeper analysis of processes during the thermal abuse tests is performed to reveal the origin of the manufacturing- and ageing-specific differences in safety behaviour.

### 3.2 Analysis of processes during thermal abuse

**Fig. 4:**
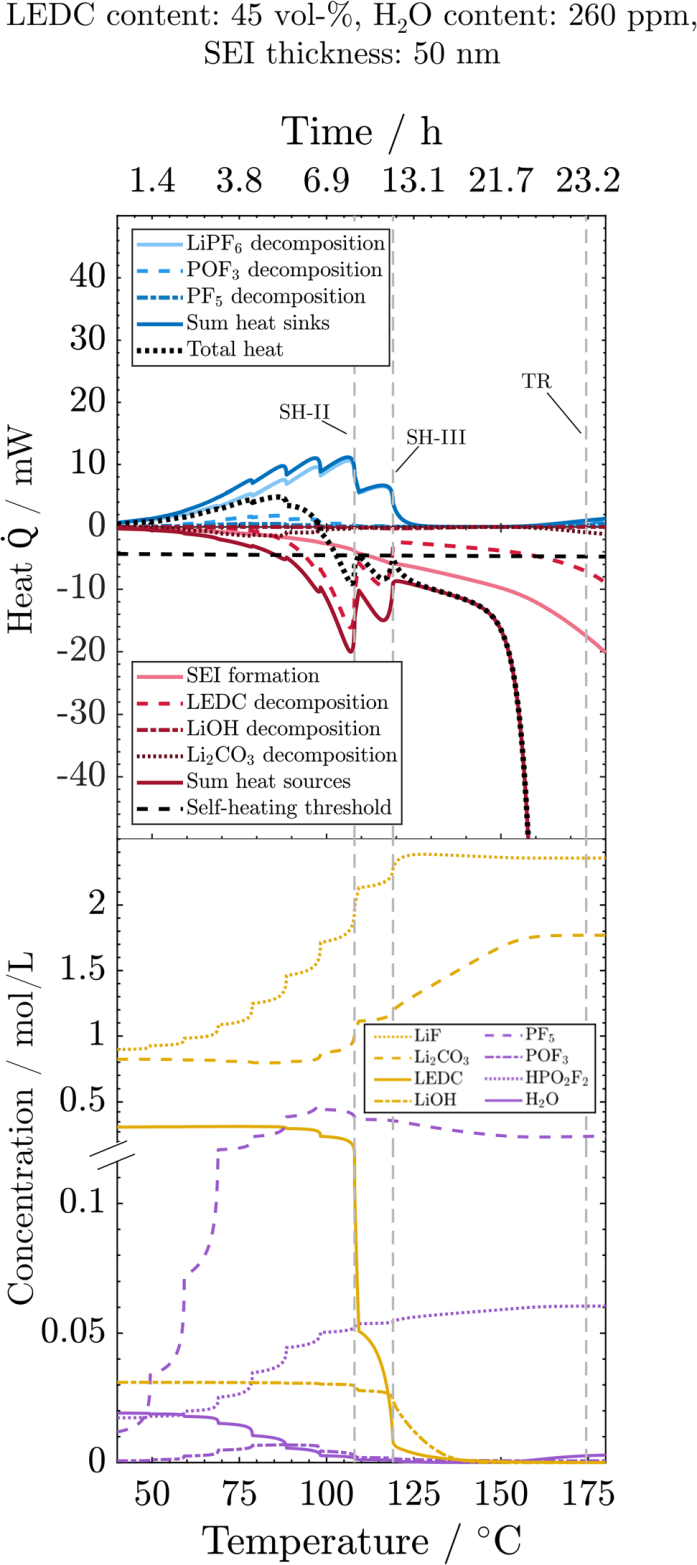
Processes during the ARC test for the reference case: evolution of heat sinks (blue) and heat sources (red) and related concentrations, as well as the total heat (black) and the self-heating threshold (horizontal dashed line). Vertical dashed lines indicate events marked in Fig. 2. The *y*-axis break is at 0.12 mol m^−3^.

To understand the main reactions impacting the self-heating and their sensitivity to the SEI state and water impurities, we here analyse the progression of reactions, related species concentrations and produced or consumed heat during the thermal misuse. For better readability, the figures display only those reactions that are substantially influenced by SEI composition or water content. For the same reason, all SEI-forming reactions, namely OSP, ISP, and LSP, are summed up. For individual contributions, see the ESI.† Analysis starts at 40 °C, where the first degradation reactions are observed (Fig. 4): the initial decomposition of the conductive salt is triggered by a shift of the equilibrium towards the decomposition product with increasing temperature. Furthermore, the decomposition of PF_5_ (PFD) and POF_3_ (POFD) starts at 60 °C, peaks around 75 °C, and ends for the first time around 110 °C (SH-II). The HF released from these reactions initiates the decomposition of Li_2_CO_3_ (ISD), which, thus, happens simultaneously to PFD and POFD. Note that the concentration of Li_2_CO_3_ is only slightly declining due to the only small amounts of H_2_O, and thus reactant HF, whereas it is produced in significant rates also from LEDC decomposition. Decomposition of PF_5_, POF_3_ and Li_2_CO_3_ happen simultaneously, and the ratio of produced heat by Li_2_CO_3_ decomposition *vs.* the consumed heat by PF_5_ and POF_3_ decomposition is always below 1 and decreases with temperature. Thus, the reactions caused by salt decomposition products, *i.e.* PFD, POFD, and ISD, act as heat sinks. The cause of the extinction of these reactions is the depletion of the necessary reactant H_2_O. As the reaction rates and, therefore, their interplay are strongly dependent on water availability, we continue the discussion when analysing the wet and dry case scenarios. Reactions connected to salt decomposition are complemented by the exothermic decomposition of LEDC with notable reaction rates occurring above 80 °C. Eventually, by increased decomposition of LEDC and already decreasing endothermic heat due to H_2_O depletion, the first self-heating starts at 108 °C (SH-II). This phase is, however, not self-sustaining because the exothermic LEDC decomposition slows down when much of the available LEDC has been consumed, so that a further heating step is required to trigger a thermal event. The transition to continuous self-heating of the battery at 118 °C (SH-III) is caused by the almost complete decomposition of LiPF_6,_ which reduces the endothermic heat to nearly 0. The self-heating phase until the rapid thermal runaway is dominated by the re-formation of the organic SEI, *i.e.* LEDC, and the inorganic SEI, *i.e.* Li_2_CO_3_, and further decomposition of LEDC. The declining concentration of LiOH denotes its decomposition to Li_2_O (LSD) around 120 °C. The amount of LiOH introduced into the system by H_2_O in the anode after drying is too small to produce notable amounts of exothermic heat. The thermal runaway is eventually set in motion by the decomposition of the cathode active material (CSD) starting around 150 °C. The thus produced O_2_ triggers the subsequent solvent combustion, first of EC (ECD) and then of EMC (EMCD); see the ESI.† The combustion product H_2_O, in turn, triggers an exponential increase in PF_5_ (PFD) and POF_3_ (POFD) decomposition reactions; despite being endothermic, they cannot compensate for the strongly exothermic reactions. Simultaneously, the exothermic decomposition of Li_2_CO_3_ is also re-initiated by HF produced from the PF_5_ and POF_3_ decomposition. Finally, it should be noted that above 130 °C, the LEDC concentration is kept at around zero, as any generated LEDC is directly consumed. In contrast, the LiF concentration increases until all LiPF_6_ is decomposed and then stays constant. The SEI is thus completely inorganic at higher temperatures. Concentrations of HF, LiPF_6_ and Li_2_O are either 0 (HF) or can be deduced from LiF (LiPF_6_) or LiOH (Li_2_O) progressions. For the sake of clarity, they are shown in the ESI.†

Having understood the process interplay for the reference case, we now evaluate how their dependence on SEI composition and thickness, and water content can explain the observed change in thermal self-heating and thermal runaway behaviour.

**Fig. 5:**
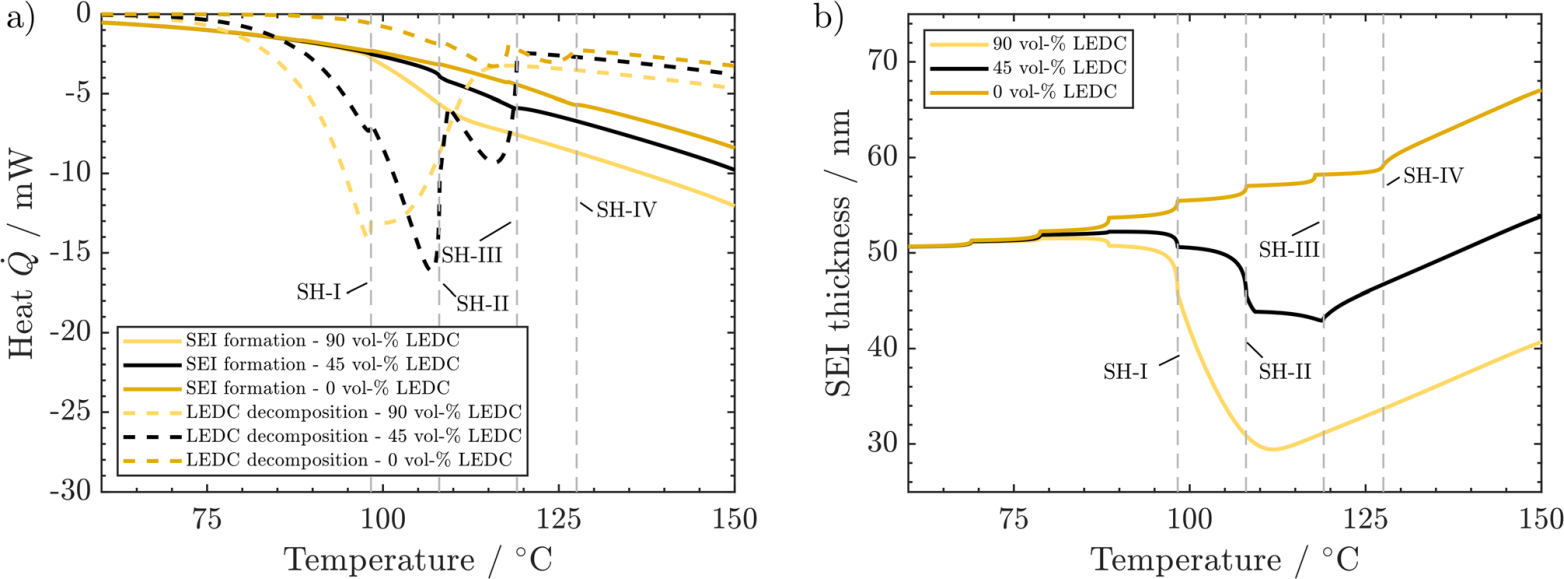
Effect of SEI composition on behaviour during the ARC test: (a) produced heat from LEDC decomposition and SEI formation. (b) Corresponding changes in SEI thickness. Conditions: 260 ppm water, 50 nm thick SEI.

As SEI composition impacts mainly the SEI formation and SEI decomposition reactions, their corresponding heat is presented in Fig. 5a. Li_2_CO_3_ decomposition is only marginally affected by changes in the SEI composition and is thus displayed together with other reactions in the ESI.†

A higher LEDC volume fraction leads to notably earlier on-set of LEDC decomposition. This leads to the earlier self-heating on-set at 98 °C (SH-I) for the 90 vol% LEDC case compared to the reference case (45 vol% LEDC). For 90% LEDC, heat production from this reaction decreases much slower after reaching self-heating because there is still a significant amount of LEDC in the SEI (see the ESI†). Together with the higher SEI formation reaction, this can explain why the 90 vol% LEDC case needs no further heating step to proceed to thermal runaway. The large fraction of LEDC in the initial SEI and its rapid but not complete consumption lead to a significant drop in SEI thickness to 60% before SEI formation sets in and rebuilds the SEI. The decline is much more significant than for the reference case, where *ca.* 80% of the SEI, mostly inorganic, remains and is subsequently rebuilt. For the inorganic case, *i.e.* 0 vol% LEDC in the initial SEI, the missing exothermic heat from the decomposition of the initial LEDC and the slow exothermic SEI formation lead to additional heating steps. At around 128 °C, marked by SH-IV, eventually, self-heating of the battery sets in due to higher exothermic SEI formation rates at this temperature. As no LEDC is present in the initial SEI in this case, and as Li_2_CO_3_ decomposition is negligible and compensated by its production, SEI thickness increases monotonously. In all three cases, during proceeding self-heating >120 °C, the heat from LEDC decomposition is smaller than that from SEI formation. It can be concluded that LEDC content in the SEI is strongly impacting thermal safety, as the self-heating onset is strongly impacted by LEDC decomposition and formation rates, and thus by LEDC availability.

**Fig. 6:**
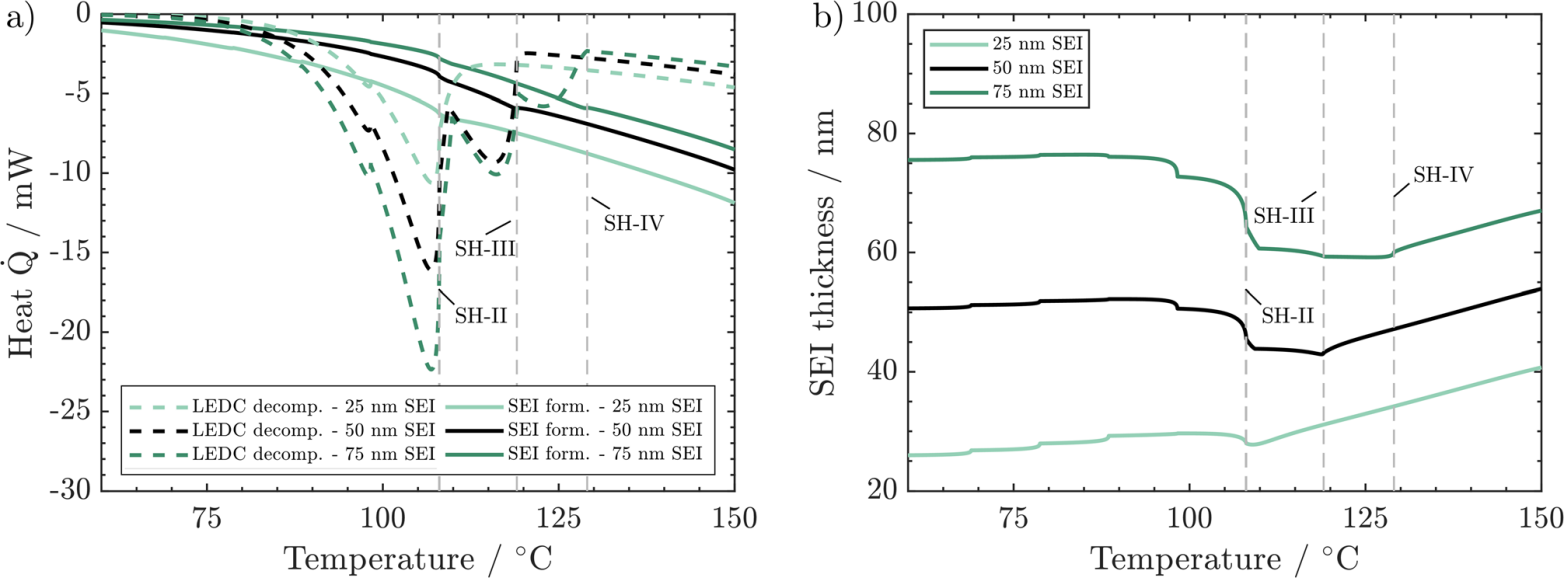
Effect of SEI thickness on behaviour during the ARC test: (a) produced heat from LEDC decomposition and SEI formation. (b) Corresponding changes in SEI thickness. Conditions: 260 ppm water, 45 vol% LEDC.

We now discuss the impact of initial SEI thickness on heat evolution from LEDC decomposition and reformation (Fig. 6a) and on the resulting changes in SEI thickness (Fig. 6b). From Fig. 2b, we know that a thicker initial SEI leads to a higher self-heating temperature. This is counterintuitive as more SEI, *i.e.*, more LEDC, means more reactants for low-temperature decomposition. Indeed, more SEI leads to more LEDC decomposition (Fig. 6a); yet, during the seek period, LEDC decomposition heat decreases to similar values for all SEI thicknesses, whereas, for the thin SEI, exothermic SEI formation is almost double that of the thicker SEIs. The thickness-dependent SEI formation rate, eqn (3), is the key to explain why a thin SEI still leads to lower self-heating temperatures. The thinner the SEI is, the higher the formation rate and thus the heat produced from SEI formation reactions. SEI thickness can therefore be seen as a beneficial property of the SEI to prevent further formation and early thermal runaway. Our findings are also in good agreement with ARC tests of cycling-aged cells in the temperature range of 35–45 °C from the literature by Feng *et al.*,^23^ Feinauer *et al.*,^22^ Börner *et al.*,^20^ and Waldmann *et al.*^21^ All studies independently found that these ageing procedures lead to an increase in the on-set temperature of self-heating by 15–20 °C when compared to their fresh reference. Our study now delivers an explanation for this increase: when cells age, the SEI becomes thicker and more inorganic; both effects have been shown here to lead to a delayed self-heating temperature. Röder *et al.*^56^ in contrast found a lowered self-heating temperature for calendaric-aged cells at 60 °C. Our model may explain this behaviour also: either the SEI had a much more total amount of LEDC, probably dissolved also in the electrolyte, or the LEDC had reacted at 60 °C and left a thin, less inhibiting SEI.

**Fig. 7:**
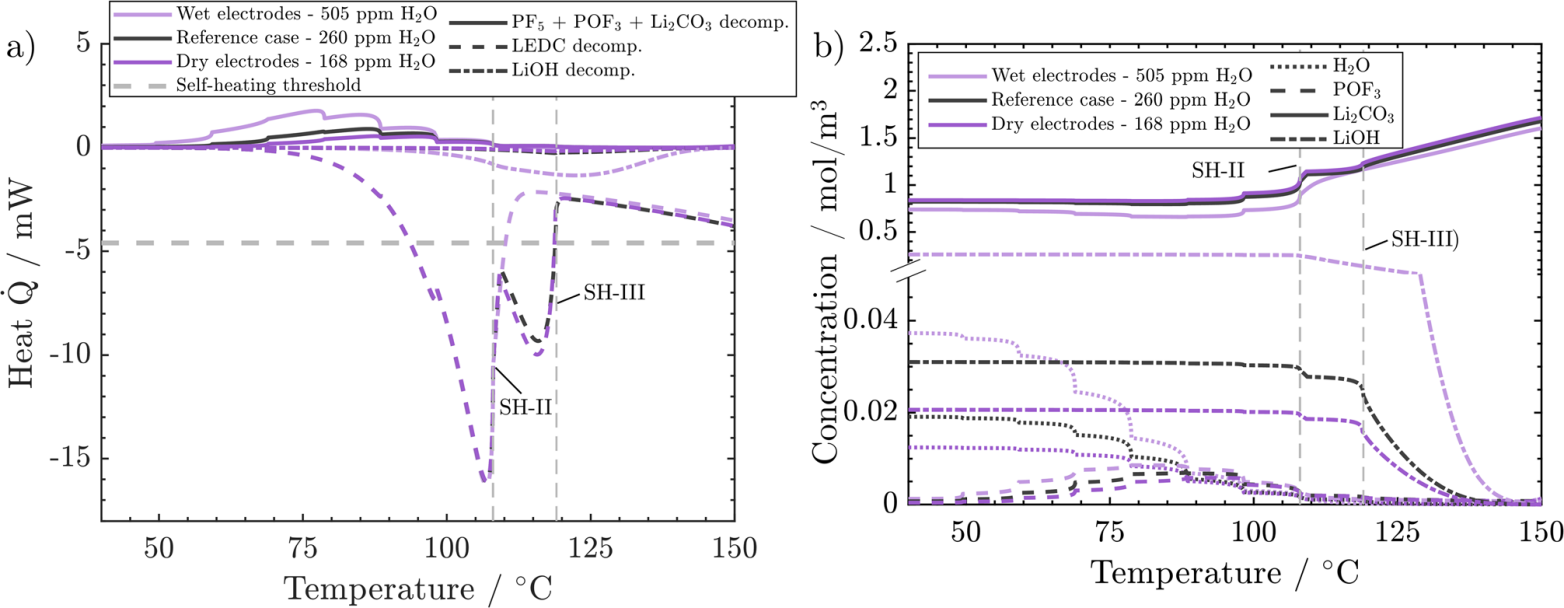
Effect of H_2_O impurities on behaviour during the ARC test: (a) combined endothermic heat of decomposition of PF_5_, POF_3_, and Li_2_CO_3_, *vs.* exothermic heat of LEDC and LiOH decomposition. (b) Concentrations of H_2_O, POF_3_, Li_2_CO_3_, and LiOH. Conditions: 45 vol% LEDC, 50 nm SEI.

Finally, we analyse the effect of H_2_O content deeper to understand why low and medium water impurities lead to the same temperature evolution while high water content leads to earlier self-heating. PF_5,_ POF_3_ and Li_2_CO_3_ decomposition reactions (PFD, POFD, and ISD) happen almost simultaneously. Their added values are endothermic and increase with water content (Fig. 7a). It can also be observed that the higher the water content, the earlier the on-set of these reactions. In the case of dry and reference H_2_O concentrations, the released heats are almost identical and very small. From *ca.* 80 °C onwards, in the wet electrode case, most of the endothermic heat is still small and released in a temperature range where no exothermic counterpart exists. With increasing impurity concentrations, the temperature gradient after sustained self-heating changes from 4.4 °C h^−1^ (dry case) to 4.65 °C h^−1^ (medium dried case) to 4.1 °C h^−1^ (wet case). These changes are connected to the increased concentration of LiOH, which decomposes exothermically, and the corresponding decrease in self-heating temperature (wet case). First, when moving from the dry case to the medium dry case, the temperature gradient only increases slightly which can be explained only by a small increase in LiOH content from 0.4 vol% to 0.6 vol%. However, in the wet case the temperature gradient decreases, which is counterintuitive at first but is also connected to the higher LiOH content of 5 vol%. The significantly higher concentration of LiOH in the wet electrode case leads to notable LiOH decomposition rates and exothermic heat in the self-heating critical temperature frame of 100–130 °C. This leads to a transition into self-heating one temperature step earlier. The lower temperature in turn leads to a slower progression of all occurring reactions and, thus, a lower temperature gradient. This points to an important characteristic of an ARC measurement. As discrete temperature steps are used, more produced heat can either increase the temperature gradient (dry → medium dry), when self-heating starts during the same time step. Or it leads to a lower self-heating temperature, with a subsequent lower temperature gradient. Thus, it is important to discuss both characteristics together. In order to illustrate these impacts, the water variation case was simulated using a 2.5 °C temperature step instead of the 10 °C used before (see the ESI†). Even though the wet case also transitions into self-heating one step earlier, the gradients, 2.5210 °C h^−1^ (dry), 2.5459 °C h^−1^ (medium) and 2.6975 °C h^−1^ (wet), now better correspond to the produced heat as the temperatures are closer together.

In conclusion, H_2_O impurities only play a significant role in battery safety when the electrodes have not been dried properly. The impact is negligible as soon as even a medium-intense drying procedure (260 ppm residual H_2_O) is applied. Also, further drying (168 ppm residual H_2_O) does not bring any noticeable benefit, because already at medium rates, LiOH decomposition rates are too low to give a significant heat contribution to tilt the balance towards sustained heating. As such, at least under the analysed circumstances and battery chemistry, extensive drying is unnecessary for a safer performance. This result aligns with the experimental findings of Huttner *et al.*,^32^ who found a negative impact on performance metrics for intense drying procedures. Two more points should be accounted for in a holistic analysis of battery safety, which may be followed in further studies: the acids produced from H_2_O, such as HPO_2_F_2_, are present in higher concentrations, *i.e.*, 0.175 mol m^−3^ in the wet case compared to 0.078 mol m^−3^ in the reference case. The acids were reported to lead to increased dissolution of transition metals from the cathode, which will then promote SEI decomposition.^57^ Eventually, even though the energetic impact of these reactions is small, all are gassing reactions and will impact cell pressure.

### 3.3 Impact of variation in the SEI and H_2_O content

So far, we have analysed how the thermal safety behaviour changes when varying a single variable, SEI thickness, composition or water content. In reality, multiple factors change due to ageing or different manufacturing processes. In the following, the impact of the cross-influence of the three variables is analysed and trends and generalisations are deduced.

**Fig. 8:**
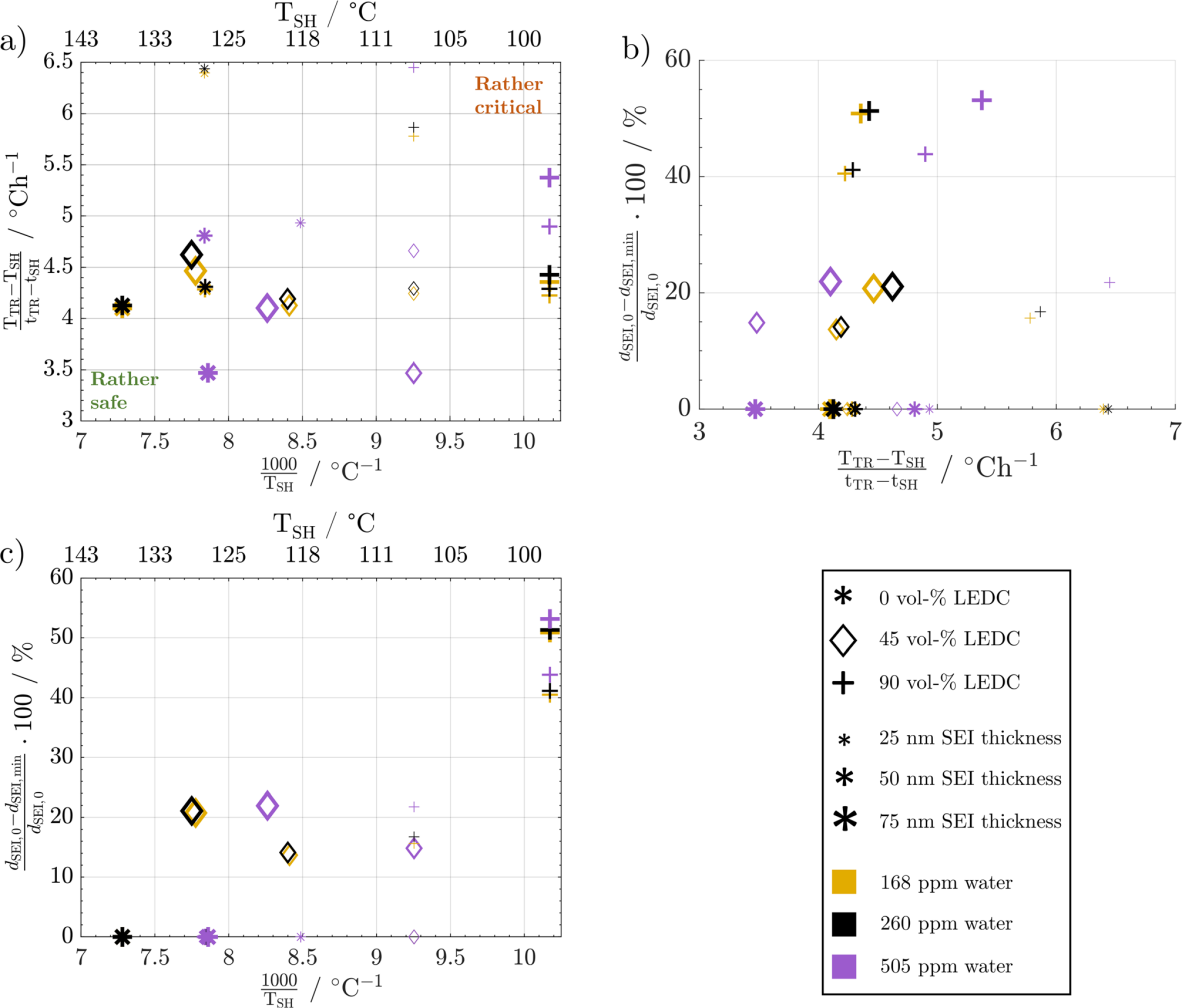
Impact of changes in water content and SEI properties on: (a) temperature gradient over the inverse self-heating temperature scaled by 1000, (b) the percentage of SEI thickness reduction over the temperature gradient, and (c) the percentage of SEI thickness reduction over the inverse self-heating temperature scaled by 1000.

Fig. 8a shows the temperature gradient in the region between self-heating on-set and thermal runaway, 
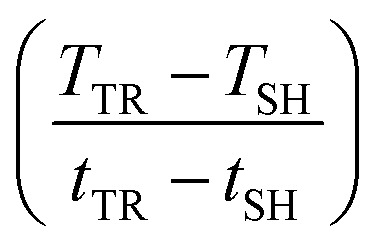
, over the inverse self-heating temperature, 
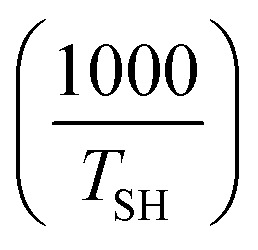
, for all 27 variations. Here, a high temperature gradient refers to a fast heating rate and, thus, a lower time to intervene. A low self-heating temperature indicates a lower resistance of the battery against thermal abuse. Thus, both characteristics can be considered indicators of battery safety and have therefore been chosen for this comparison. Cases that are located in the lower left corner of the figure can be considered rather safe, because it represents high self-heating temperatures and a low self-heating rate, whereas, the cases closer to the upper right show the opposite characteristics and, thus, can be considered rather critical. There are cases in all four quadrants of the figure, so no general correlation between the cases with fast self-heating and self-heating temperature can be found. The graph shows that the highly critical variations almost exclusively include high LEDC contents and a thin to medium thick SEI. The safe region on the other hand embodies almost exclusively all thick SEI variations with a large proportion of the inorganic cases. We can also observe that the thin SEI cases tend to have high temperature gradients probably due to the faster reformation at higher temperature. Higher LEDC content correlates with lower self-heating temperatures, as LEDC decomposition already occurs at low temperatures, whereas Li_2_CO_3_ decomposition is less strong at these temperatures. Variations of H_2_O are scattered over the whole figure, which indicates that its influence is not as significant and straight-forward as the SEI properties even for high contaminations. This confirms the lower sensitivity to H_2_O content than to SEI thickness and LEDC content.

The complex water effects warrant further analysis. For cells with thick inorganic SEIs (large stars, Fig. 8a) the wet case (violet) has a lower self-heating temperature and a lower gradient compared to the dry and medium dry case. This means that it enters the self-heating phase earlier but then heats up slower, *i.e.* one metric gets worse and the other gets better. In contrast, for the high LEDC contents (+) the higher water contamination value does not lead to lower self-heating temperature and the temperature gradient shows the opposite behaviour to the inorganic case: it increases. The root cause lies in the onset of LiOH decomposition. Only in cases of high water contamination is the LiOH amount high enough to significantly impact the temperature progression, which we showed in Fig. 7a. The onset of this exothermic decomposition is around >115 °C, which is higher than the self-heating temperature of all high LEDC cases. This means that the LEDC cases are already in sustained self-heating when the LiOH decomposes; thus, water content does not impact the self-heating temperature for high LEDC content cases, whereas for all cases that have not transitioned to sustained self-heating before LiOH decomposes, the heat from LiOH decomposition impacts the self-heating temperature: the higher the water content and thus LiOH concentration, the more LiOH decomposition heat, thus the lower the self-heating temperature. The observed lower temperature gradient for the wet, inorganic and reference LEDC concentration cases is directly correlated with the lower temperature itself. The lower the temperature the slower all reactions progress, and thus, the less heat is produced.

A low SEI thickness has been shown to accelerate the exothermic SEI reformation; thus, we analyse for the different cases how much the SEI is reduced and how this correlates to the temperature gradient (Fig. 8b) and the self-heating temperature (Fig. 8c). The high LEDC content cases (+) exhibit the highest decline in SEI thickness compared to the medium and inorganic cases. The low thicknesses for high LEDC contents in turn accelerate the reformation and, thus, contribute to higher temperature gradients. The high concentration of LEDC also leads to a low self-heating temperature. The pure inorganic cases, represented by stars, and most of the thin SEI cases, represented by small symbols, do not show any thickness reduction. Inorganic SEI cases do not show a decrease in SEI thickness because the decomposition of Li_2_CO_3_ is not substantial enough to compensate for the SEI formation rates even for the thick SEI cases. Therefore, no decrease in SEI thickness can be observed. For the thin SEI cases, the thickness does not decrease substantially because the thin SEI accelerates SEI reformation, which counteracts SEI decomposition reactions. Most of the thin SEI and the inorganic SEI cases show a correlation between the temperature gradient, the self-heating temperature and the SEI thickness: the thinner the SEI the higher the temperature gradient and the lower the self-heating temperature. For the high LEDC content and thin SEI cases, the high amount of LEDC decomposes before substantial reformation starts. Thus, they show a slight SEI thickness reduction. Due to their anyway thin SEI thickness they also show the highest temperature gradient among all organic cases. Comparing the thin SEI cases with the medium thick SEI cases it shows that the medium thick SEI cases exhibit lower temperature gradients and higher self-heating temperatures. This is explained by the accelerated reformation rates for the thin SEI cases, shereas, the comparison of the medium thick SEI and thick SEI cases reveals that the thick SEI cases have a higher temperature gradient. This is explained by the higher self-heating temperature of the thick SEI cases. Since they transition at higher temperatures into the self-heating phase, the reactions occur faster and more heat is produced.

From these extensive variations and their impact on thermal safety behaviour, it can be concluded that the SEI thickness and LEDC content are the dominant effects in terms of battery safety. A safer battery has an inorganic, thick SEI. Safety decreases with increasing LEDC content and reducing SEI thickness. Besides these two, H_2_O impurities only play a role when severe contamination is present and generally contribute less than the SEI properties. The effect of water is also more challenging to address. High contamination affects the self-heating temperature and the temperature gradient differently depending on the composition of the SEI.

## 4 Conclusion

This study has elucidated the impact of the initial SEI state and water impurities on the thermal safety behaviour of Li-ion batteries with EC:EMC 1.2 M LiPF_6_ and a graphite anode, including when and why self-heating occurs and the subsequent progression to rapid thermal runaway. Initial concentrations of SEI components, impurities and conductive salt decomposition products were rigorously derived from assessing manufacturing, production and ageing effects.

Dominant detrimental effects are high LEDC concentrations and a thin SEI, such as those found in rather fresh cells. Here, a high LEDC content could be connected to an earlier onset of self-heating. In contrast, a thinner SEI relates to faster SEI reformation and thus to a higher temperature gradient. The experimentally observed increase in self-heating temperature for aged cells^20–23^ is thus attributed to an ageing-induced change from a foremost organic SEI to an inorganic SEI and a thicker SEI, which delays exothermic SEI reformation processes. The impact of H_2_O impurities on battery safety is found to be marginal as long as a moderate drying procedure is applied to the electrodes during manufacturing. Thus, we could show that extreme electrode drying does not benefit battery safety. However, high H_2_O contamination during production should be avoided as this will have a substantial negative impact. Here, the effect of high contamination was found to depend on the SEI composition. For an inorganic and mixed SEI, the contamination will reduce the self-heating temperature due to decomposition of LiOH. High LEDC content cases, on the other hand, exhibit a higher temperature gradient, because here the self-heating already starts before LiOH decomposition sets in.

The insights gained here contribute significantly to understanding and controlling Li-ion battery behaviour during thermal abuse. The trends for impact of water as an electrolyte impurity, the complexities of SEI properties, and their combined battery safety have been shown, and they cross influence each other. The presented degradation reactions and kinetics are suitable for integration into full cell models to evaluate the impact of local hotspots and heat removal, and thus to reveal battery runaway and propagation on cell and pack levels. The studies may be further extended to include the effects of different active materials and electrolytes, as reactions, reactivity and mechanical stability may change. Different experimental behaviours were reported here, especially for the highly reactive Li metal and solid state electrolytes. Also of special importance is the interaction of the cathode with water impurities and of metal dissolution on the reaction network and the thermal safety behaviour.

## Abbreviations

*a*
: Activity (—)
*α*
: Dissociation degree (—)
*C*_p,bat_
: Battery heat capacity (J K^−1^)
*d*_SEI_
: SEI thickness (m)
*E*_A_
: Activation energy (J mol^−1^)
Δ_r_*G*: Gibbs free energy of reaction (J mol^−1^)
Δ_r_*H*_*j*_: Enthalpy of reaction (J mol^−1^)
*k*_f/b_
: Frequency factor forward/backward reaction (mol s^−1^, (mol m) s^−1^)
*K*
: Equilibrium constant (—)
*ν*
: Stoichiometric coefficient (—)
*n*
: Molar amount (mol)
*r*
: Reaction rate (mol s^−1^)
R: Molar gas constant (J (mol^−1^ K^−1^))
Δ_r_*S*: Entropy of reaction (J (mol^−1^ K^−1^))
*t*
: Time (h)
*t*_SH_
: Self-heating time (h)
*t*_TR_
: Thermal runaway time (h)
*T*
: Temperature (K, °C)
*T*_SH_
: Self-heating temperature (K, °C)
*T*_TR_
: Thermal runaway temperature (K, °C)

## Data availability

Source data are provided with this paper in the KITopen repository under https://doi.org/10.35097/1804.

## Author contributions

Florian Baakes: conceptualisation, methodology, software, writing –original draft, validation, investigation, visualisation. Daniel Witt: conceptualisation, writing – review & editing. Ulrike Krewer: conceptualisation, writing – review & editing, supervision, funding acquisition.

## Conflicts of interest

There are no conflicts to declare.

## Supplementary Material

SC-014-D3SC04186G-s001
