# Supplementary material for: Impact of electrolyte impurities and SEI composition on battery safety
Source: Chem Sci. 2023 Nov 3;14(47):13783–98. doi: 10.1039/d3sc04186g (PMC10699578; doi:10.1039/d3sc04186g)
Supplement: SC-014-D3SC04186G-s001 [file SC-014-D3SC04186G-s001.pdf]

Electronic Supplementary Material (ESI) for Chemical Science.
This journal is © The Royal Society of Chemistry 2023

Electronic Supplementary Information

Impact of electrolyte impurities and SEI composition on battery safety

Florian Baakes, Daniel Witt, Ulrike Krewer

Content

1. Preamble 3
2. Reference conditions 3
3. Estimation of initial values of $LiPF_6$ decomposition products 4
4. Estimation of initial SEI composition 7
5. Additional model equations 8
6. Initial values 9
6 Kinetic parameters 13
7 Additional figures 14
References 20

1. Preamble

The following document contains the information needed to replicate the thermal runaway study of Li-ion batteries presented in the main manuscript, if it is not given in our earlier publication [1]. Also, figures and descriptions are added that make it easier to understand the performed study or how certain parameters have been obtained.

2. Reference conditions

Using the reactions connected to conductive salt decomposition, namely CSD, PFD and POFD, we can estimate the initial values for their reactants. Here, we emulate the initial mixing of the electrolyte by a simulation of the reaction of electrolyte species with 40 ppm H_2O, which is present as an impurity in the electrolyte. At equilibrium, most of the water has been consumed, and the electrolyte contains 8 ppm H_2O and 50 ppm HF which is in excellent agreement with the recorded <10 ppm H_2O and <50 ppm HF by electrolyte suppliers[2]. During cell assembly, we assume that further H_2O is introduced into the system, originating from the separator and both electrodes. Values for water contamination for the anode, cathode, and separator are based on the medium drying procedure reported in the publication by Huttner et al.[3]. This is estimated to result in an additional 334 ppm of water in the electrolyte for the reference case. The water contained in the anode is assumed to react to LiOH fully (LSP). After cell assembly, cell formation is conducted. This procedure introduces Li_2CO_3 as part of the SEI into the system. Li_2CO_3, in turn, reacts swiftly already at room temperature with HF to form H_2O (ISD). The H_2O is then used up by the reactions of PF_5 (PFD) and POF_3 (POFD), which occur steadily but slowly at room temperature. Therefore, the last step to get realistic initial conditions for the simulation of the ARC measurement is estimating the time between cell formation and testing. For our study, we assume direct testing and consider the initial values after a formation period of three days with C/10. Details and intermediate results are described in the below. This procedure calculates the initial values for the electrolyte decomposition products PF_5, POF_3, HPO_2F_2, HF, and H2O as 992 ppm, 46 ppm, 1306 ppm, 0 ppm, and 260 ppm, respectively.

Initial values for the SEI composition are challenging to obtain as most literature does not report quantitative values. Thus, we base our assumptions on the study of Liu et al. [4]. Using an electrochemical quartz crystal microbalance, they measured the mass increase on a graphite anode during formation. Combined with electrochemical data, online electrochemical mass spectrometry measurements, and x-ray photoelectron spectroscopy, they could reasonably estimate the corresponding mass attributed to individual SEI species. From this, we deduct the volumetric SEI composition as follows (details see below):

- Li_2O: Liu et al.[4] found that within the first discharge after formation, the anode lost weight. Based on the measured weight loss, they assume the following oxidation to occur: $(CH_2OCO_2Li)_2 \rightarrow Li_2O + 2CO_2 + C_2H_4 + O_2$. Following this, we estimate the Li_2O content to be 10 vol-%.
- $(CH_2OCO_2Li)_2$: The content is estimated to be 45 vol-%
- LiOH: It is assumed that all residual water in the anode reacts to form LiOH (LSP) during cell formation. This amounts to 0.6 vol-% for the reference wetting case.
- LiF: Since Liu et al.[4] could not distinguish between LiOH and LiF, we assume that both substances add up to 10 vol-% of the SEI. Combined with the additional production of LiF by decomposition of Li_2CO_3 (ISD) during the applied ageing procedure, this amounts to 10.4 vol-% for LiF.
- Li_2CO_3: The last 34 vol-% of the SEI is assumed to be composed of Li_2CO_3.

3. Estimation of initial values of $LiPF_6$ decomposition products

In the following, the results including a visualization for the procedure of identifying proper initial values for the decomposition products of $LiPF_6$ are shown.

In Figure 1, the initial mixing of the electrolyte is shown. The very first process occurring is the equilibration of $LiPF_6$ with PF_5 and LiF. This process happens extremely fast. Afterwards, PF_5 and POF_3 decompose with the water within the solvent. After 60 days, the H_2O concentration dropped under 10 ppm, and the HF concentration rose over 50 ppm. This is in very good agreement with the <15 ppm H_2O

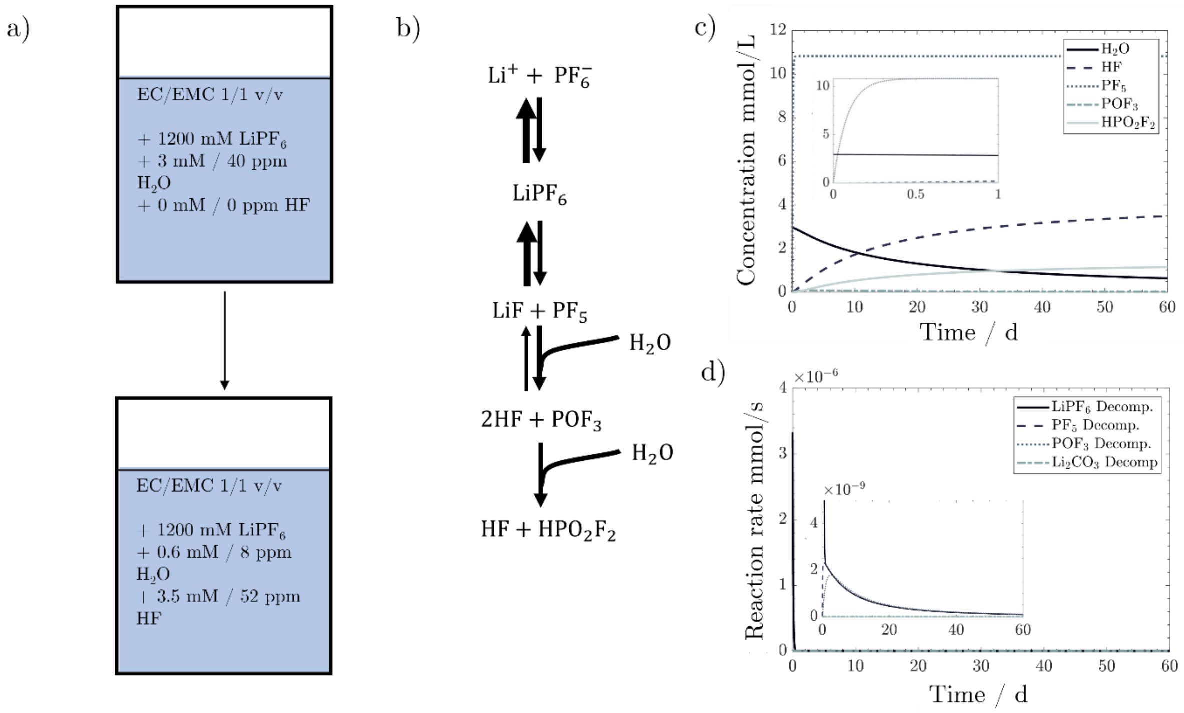

Figure 1: Calendaric aging of the electrolyte after initial mixing with a) initial and final values after 60 days for $LiPF_6$, H_2O and HF, b) the considered reaction scheme. c) the concentration progression and d) the reaction rates.

and <50 ppm HF reported by electrolyte producers [2].

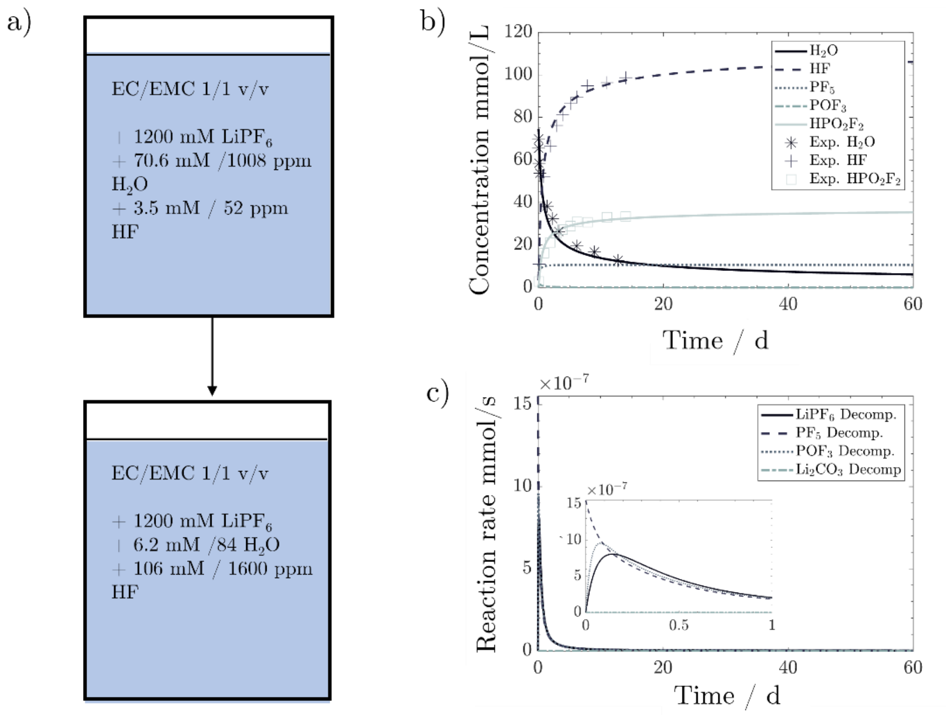

Figure 2: Calendaric aging of electrolyte after adding 1000 ppm of water to initial electrolyte mixture with a) initial and final values after 60 days for $LiPF_6$, H_2O and HF, b) the concentration progression with experimental values taken from Stich et al. and c) the reaction rates.

Figure 2 shows the same system after adding 1000 ppm or 70 mM of H_2O as an impurity. The results resemble those of the experimental study conducted by Stich et

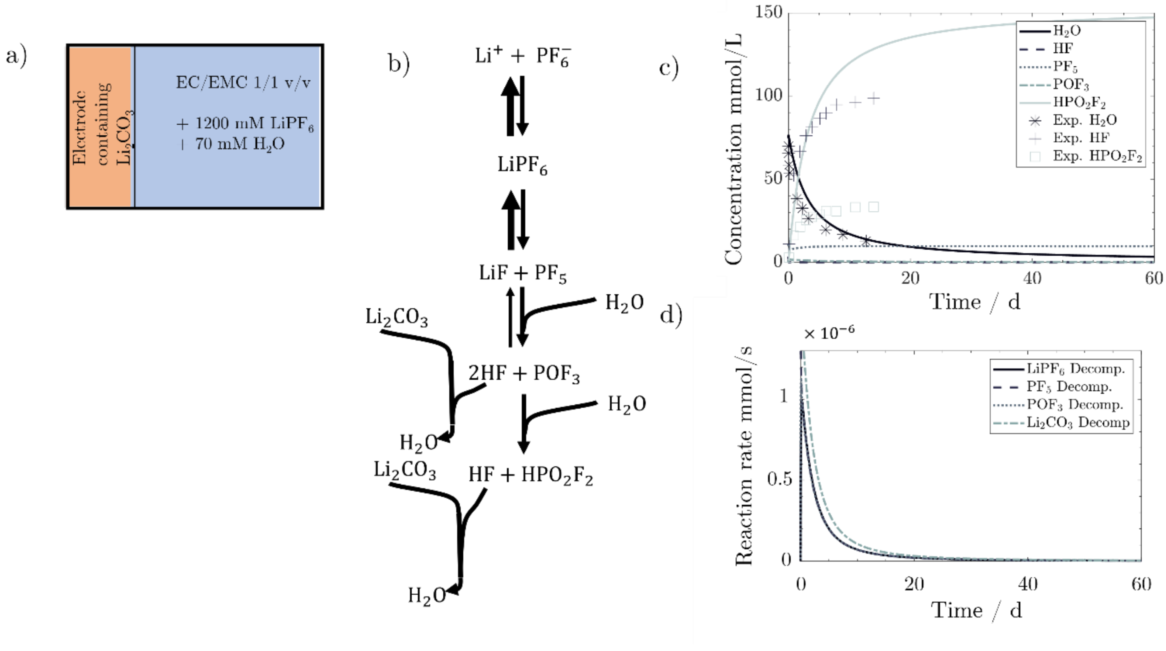

Figure 3: Simulation of impact of cell assembly, formation and subsequent 60 days calendaric ageing on battery composition with a) sketch of the simulated setup, b) the considered reaction scheme, and c) the concentration progression with experimental values taken from Stich et al. as comparison to reactions without Li_2CO_3, and d) the reaction rates.

al., which was used to parameterize the reactions shown in Figure 2 b). Experimental points, extracted from Stich's publication [5], are shown as stars, crosses, and circles, referring to HF, H_2O, and HPO_2F_2, respectively.

In Figure 3, the change occurring in the "formation and conditioning" period is shown, and our simulation including Li_2CO_3 is compared to the experimental results of Stich et al.[5] where no Li_2CO_3 was present. Due to very fast reaction of Li_2CO_3 with HF, the HF level almost immediately drops to 0. Further, due to the presence of H_2O, the depletion is slowed down and the concentration of HPO_2F_2 is drastically increased. Based on the strong difference between the electrolyte composition before and after assembly and formation, we conclude that it is advised to conduct studies on electrolyte ageing always in a true battery environment, if the goal is to compare electrolyte composition of a fresh cell with aged cells.

4. Estimation of initial SEI composition

In the following, the calculations for the initial SEI composition are presented. In their study, Liu et al.[4] measured a maximum SEI weight of 3250 ng. 1750 ng are attributed to LEDC based on the slope of 80 $\Delta g/\Delta C$ mass change per charge. Here, the value has to be multiplied by two since two electrons are transferred to produce LEDC. The resulting 160 $\Delta g/\Delta C$ are very close to molar mass of 162 g/mol for LEDC. In the first discharge, the overall weight is reduced to 2400 ng. Here, the re-oxidation of LEDC is assumed to take place following the reaction: $(CH_2OCO_2Li)_2 \rightarrow Li_2O + 2CO_2 + C_2H_4 + O_2$. This results into a weight loss of 850 ng. Considering a 1.22 ratio of weight of the gaseous products to LEDC, a loss of 1040 ng LEDC is estimated and a production of roughly 200 ng Li_2O. Considering the overall mass of the SEI of 2400 ng and 700 ng of LEDC and 200 ng of Li_2O, translates to 32 wt-% LEDC and 8 wt-% Li_2O. Further, 350 ng LiF production translates to 14 wt-% of SEI content. This corresponds to 45 vol-%, 8vol-% and 12 vol-% for LEDC, Li_2O and LiF, respectively. The amount of LiOH is calculated based on the assumption that all H_2O present in the anode after drying will react to LiOH. For the reference case, this gives 0.6 vol-% LiOH. We round Li_2O and the sum of LiF and LiOH to 10 vol-% each. Eventually, the last 33 vol-% are

assumed to consist of Li_2CO_3. Please note that these estimations give only a rough idea of the order of magnitude for the SEI composition. Also note that these are the values before the “formation and conditioning” procedure, described above, will be performed. Thus, the initial compositions listed in Table 2 are slightly different to the ones described here.

5. Additional model equations

Table 1: Constitutive model equations for the simulation of a thermal abuse in Li-ion batteries

Description	Equation	No.
Energy balance	Preheating: $\frac{\mathrm{dT}}{\mathrm{dt}} = 1\frac{°\mathrm{C}}{\mathrm{min}}$ Heat − Wait − Seek: $\frac{\mathrm{dT}}{\mathrm{dt}} = \frac{\mathrm{T_{Setpoint}} - \mathrm{T}}{\mathrm{K}}$	(1), (2)
Heat capacity	$\mathrm{C_{p,bat}} = \sum_{i\in\Omega_{\mathrm{SP}}} \mathrm{c}_{\mathrm{p},i}\, \mathrm{n}_i + \mathrm{C_{p,Al}} + \mathrm{C_{p,Cu}} + \mathrm{C_{p,Casing}} + \mathrm{C_{p,Separator}}$	(3)
Produced heat	$\mathrm{q}_j = \mathrm{r}_j \Delta_{\mathrm{r}} \underline{\mathrm{H}}_j(\mathrm{T}), \forall j \in \Omega_{\mathrm{RE}}$	(4)
Activity, solubility	$\mathrm{C}_i = \frac{\mathrm{n}'_i}{\mathrm{V}}, \forall i \in \Omega_{\mathrm{SP}}$ $\mathrm{p}_g = \mathrm{p}\mathrm{x}''_g\ \forall g \in \Omega_{\mathrm{G}}$	(5), (6)
	$\mathrm{a}_l = \frac{\mathrm{C}_l}{\mathrm{C}^{\Theta}}, \forall l \in \Omega_{\mathrm{L}}$ $\mathrm{a}_s = \frac{\mathrm{C}_s}{\mathrm{C}^{\Theta}}, \forall s \in \Omega_{\mathrm{S}}$ $\mathrm{a}_g = \frac{\mathrm{p}_g}{\mathrm{p}^{\Theta}}, \forall g \in \Omega_{\mathrm{G}}$	(7), (8), (9)
	$\mathrm{x}'_{g,\sigma} = \frac{\mathrm{p}_g}{\mathrm{H}_{g,\sigma}} \forall\ g \in \Omega_{\mathrm{G}} \setminus \{\mathrm{EC,EMC}\}\ \wedge \forall \sigma \in \{\mathrm{EC,EMC}\}$	(10)
	$\mathrm{n}'^{,\max}_g = \sum_{\sigma\in\{\mathrm{EC,EMC}\}} \frac{\frac{\mathrm{n}'_\sigma \mathrm{p} \mathrm{n}_g}{\mathrm{H}_{g,\sigma}\sum_{\gamma\in\Omega_{\mathrm{G}}}\mathrm{n}_\gamma}}{\frac{1-(\mathrm{n}'_\sigma \mathrm{p} \mathrm{n}_g)}{\mathrm{H}_{g,\sigma}\sum_{\gamma\in\Omega_{\mathrm{G}}}\mathrm{n}_\gamma}}, \forall g \in \Omega_{\mathrm{G}} \setminus \{\mathrm{EC,EMC}\}$	(11)
	$\mathrm{n}'_g, \mathrm{n}''_g = \left\{ \begin{matrix} \mathrm{n}'_g = \mathrm{n}_g, \mathrm{n}''_g = 0, \text{ for } \mathrm{n}_g \le \mathrm{n}'^{,\max}_g \\ \mathrm{n}'_g = \mathrm{n}'^{,\max}_g, \mathrm{n}''_g = \mathrm{n}_g - \mathrm{n}'^{,\max}_g \text{ for } \mathrm{n}_g > \mathrm{n}'^{,\max}_g \end{matrix} \right\} \forall g \in \Omega_{\mathrm{G}}$	(12)

6. Initial values

The full set of initial conditions of the reference case as well of the broad set of all possible combinations of cases is given in Table 2 and Table 3.

Table 2: Values for initial conditions for SEI composition and $LiPF_6$ decomposition products for all simulated variations. d_{SEI} denotes SEI thickness, ϵ the volume-fraction inside the SEI, and C_{xy} the concentration in the electrolyte.

Cases	d_{SEI}	ϵ_{LEDC}	$\epsilon_{Li_2CO_3}$	ϵ_{LiF}	ϵ_{Li_2O}	ϵ_{LiOH}	C_{H_2O}	C_{HF}	C_{PF_5}	C_{POF_3}	$C_{HPO_2F_2}$	C_{LiPF_6}
Unit	nm	vol%	vol%	vol%	vol%	vol%	ppm	ppm	ppm	ppm	ppm	ppm
R/OS/W	50	91	0	4	0	5	503	0	879	116	5358	133367
R/OS/D	50	90	9.2	0.4	0	0.4	168	0	1016	37	536	135084
R/IS/W	50	0	36	29	30	5	504	0	880	95	5348	128966
R/IS/D	50	0	39.6	30	30	0.4	168	0	1017	30	537	135092
TkS/R/W	75	45	32	8	10	5	505	0	880	95	5334	128983
TkS/R/D	75	45	34,6	10	10	0,4	168	0	1016	30	535	135093
TnS/R/W	25	46	26	13	10	5	504	0	880	95	5342	128975
TnS/R/D	25	45	34.6	10	10	0.4	168	0	1016	30	538	135091
TkS/OS/R	75	90.2	8.6	0	0.6	0.6	260	0	992	55	1319	134083
TkS/IS/R	75	0	39.4	30	30	0.6	259	0	992	46	1303	134113
TnS/OS/R	25	90.6	7	1.8	0	0.6	259	0	992	55	1319	134083
TnS/IS/R	25	0	38	31.4	30	0.6	259	0	991	46	1310	134106
TkS/OS/W	75	90.8	1.8	2.4	0	5	503	0	880	116	5363	129921
TkS/OS/D	75	90	9.2	0.4	0	0.4	168	0	1017	37	540	135079
TkS/IS/W	75	0	37	28	30	5	504	0	880	95	5349	128964
TkS/IS/D	75	0	39.6	30	30	0.4	168	0	1016	30	538	135091
TnS/OS/W	25	91	0	5	0	5	299	725	977	31	3946	130750
TnS/OS/D	25	90.2	8.6	0	0.8	0.4	168	0	1017	37	540	135082
TnS/IS/W	25	0	31	34	30	5	505	0	880	95	5344	128972
TnS/IS/D	25	0	39.1	30.1	30.4	0.4	168	0	1017	30	538	135093

R=Reference depending on position, i.e., SEI thickness, SEI composition or humidity

TkS = Thick SEI, TnS = Thin SEI, IS = Inorganic SEI, OS = Organic SEI, W = Wet, D = Dry

Table 3: Initial molar amounts ($n_i(t=0)$) for all considered species, states of each species and reference volume for concentration calculation for the reference case (45 vol-% LEDC, 260 ppm H_2O, 50 nm thick SEI).

Species	States /Sets	$n_i(t=0)$ in mmol	Reference volume
$(CH_2OCO_2Li)_2$(LEDC)	Solid/ Ω_S, Ω_{SEI}	0.5976	$V_{SEI} + V_{AM,\,An}$
Li_2CO_3	Solid/ Ω_S, Ω_{SEI}	1.5946	$V_{SEI} + V_{AM,\,An}$
$LiOH$	Solid/ Ω_S, Ω_{SEI}	0.0601	$V_{SEI} + V_{AM,\,An}$
Li_2O	Solid/ Ω_S, Ω_{SEI}	1.1143	$V_{SEI} + V_{AM,\,An}$
LiF	Solid/ Ω_S, Ω_{SEI}	1.7359	$V_{SEI} + V_{AM,\,An}$
$LiPF_6$	Liquid/ Ω_L	2.6035	V_{El}
PF_5	Liquid, Gas/ Ω_L, Ω_G	0.0232	V_{El}
POF_3	Liquid, Gas/ Ω_L, Ω_G	0.0016	V_{El}
HPO_2F_2	Liquid/ Ω_L	0.0381	V_{El}
H_2O	Liquid, Gas/ Ω_L, Ω_G	0.0424	V_{El}
HF	Liquid, Gas/ Ω_L, Ω_G	0	V_{El}
CO_2	Liquid, Gas/ Ω_L, Ω_G	0	V_{El}
O_2	Liquid, Gas/ Ω_L, Ω_G	0	V_{El}
H_2	Liquid, Gas/ Ω_L, Ω_G	0	V_{El}
Li_zCoO_2	Solid/ Ω_S	27.3571	$V_{AM,\,Cat}$
CO_3O_4	Solid/ Ω_S	0	$V_{AM,\,Cat}$
LiC_6	Solid/ Ω_S	24.6048	$V_{AM,\,An}$
$C_3H_4O_3$ (EC)	Liquid/ Ω_L	16.8104	V_{El}
$C_4H_8O_3$ (EMC)	Liquid/ Ω_L	10.2377	V_{El}

Physical parameters of species and geometrical parameters of the battery

Table 4: Property values for the added species in this publication, comprising the density, molar heat capacity, heat of formation, entropy and molar mass at standard conditions Θ of 25 °C and 101325 Pa.

Species	$\rho^{\ominus}$ kg/m^3	$\underline{c}_p^{\ominus}$ J/mol/K	$\Delta_f H^{\ominus}$ kJ/mol	$s^{\ominus}$ J/mol/K	M g/mol
$(CH_2OCO_2Li)_2$	1300[6,7]	200.82[8]	-1370.00[9]	88.8[9]	161.95[10]
Li_2CO_3	2110[11]	96.27 [10,12]	-1216.04[10]	90.31[10]	73.89[10]
Li_2O	2013[10]	54.10[10]	-598.73[10]	37.8482[10]	29.881[10]
$LiOH$	1450[10]	49.57[10]	-484.93[10]	42.8143[10]	23.94[10]8
LiF	2640[11]	41.89[10,12]	-616.93[10]	35.73[10]	25.94[10]
$LiPF_6$	1500[11]	151.15[13]	-2296.00[13]	160.8184[13]	151.91
LiC_6	1622[14]	49.22[14]	-3.9170[15]	-	79.01[10]
Li_xCoO_2	3115[14]	66.37[16–18]	-499.76[16–18]	-	97.87[10]
Co_3O_4	6110[11]	123.17[10,12]	-910.02[10]	114.44[10]	240.80[10]

C_2H_4	1.14[19]	43.13[12,19]	52.47[19]	219.32[10]	28.10[10]
H_2	0.0813[19]	28.83[19]	0[19]	107.7091[19]	2.0159[10]
CO_2	1.78[19]	37.44[12,19]	-393.52[10]	213.79[10]	44.01[10]
O_2	1.29[19]	29.43[12,19]	0[10]	205.15[10]	32.00[10]
H_2O	997[19]	75.33[12,19]	-285.83[10]	69.95[10]	18.05[10]
HF	0.83[20]	29.14[12,19]	-272.55[10]	173.78[10]	20.01[10]
PF_5	5.13[21]	84.79[10,12]	-1594.41[10]	300.80[10]	125.97[10]
POF_3	4.26[21]	68.79[10,12]	-1254.25[10]	285.41[10]	103.97[10]
HPO_2F_2	1583[22]	145.04[10]	-971.00[23]	193.0196[23]	101.977[10]
$C_3H_4O_3$	1333[24,25]	123.62[9,26]	-590.90[10]	132.54[10]	88.06[10]
$C_4H_8O_3$	1060[21,24]	175.15[24]	-645.73[10]	-	101.10[10]

Table 5: Structural parameters for thermal runaway modelling of Li-ion battery.

Parameter	Symbol	Value
Battery thickness	d_{Cell}	6.5 mm[27]
Battery length	l_{Cell}	42.5 mm[27]
Battery width	w_{Cell}	25.7 mm[27]
Cell area	A_{Cell}	1097 mm^2 *
Volume fraction AM anode	$\epsilon_{AM,An}$	0.62 [28]
Volume fraction AM cathode	$\epsilon_{AM,Cat}$	0.61 [28]
Volume fraction electrolyte anode	$\epsilon_{El\ An}$	0.32 [28]
Volume fraction electrolyte separator	$\epsilon_{El\ Sep}$	0.5 [28]
Volume fraction electrolyte cathode	$\epsilon_{El\ Cat}$	0.33 [28]
Volume fraction PVDF	ϵ_{PVDF}	0.06 *
Particle radius anode	r_{An}	1 µm [29]
Particle radius cathode	r_{Cat}	1 µm [29]
Thickness anode	d_{An}	77 µm [28]
Thickness separator	d_{Sep}	25 µm [28]
Thickness cathode	d_{Cat}	68 µm [28]
Volume cell	V_{Cell}	7.1 mL*
Volume copper	V_{Cu}	0.19 mL*
Volume aluminum	V_{Al}	0.37 mL*
Volume PVDF	V_{PVDF}	0.32 mL*
Volume pouch foil	$V_{Pouch\ foil}$	0.95 mL*
Volume AM anode	$V_{AM,An}$	1.77 mL*
Volume AM cathode	$V_{AM,Cat}$	1.54 mL*
Volume SEI	$V_{SEI,0}$	0.16 mL*
Volume electrolyte	$V_{El,0}$	1.54 mL*
Specific surface area anode	$a_{s,An}$	1860000 m^{-1}*
Specific surface area cathode	$a_{s,Cat}$	1830000 m^{-1}*
Number of double coated sheets anode	$n_{DCS,An}$	17 [28]

Number of double coated sheets cathode	$n_{DCS,Cat}$	16 [28]
Number of single coated sheets cathode	$n_{SCS,Cat}$	2 [28]
Thickness aluminum single coated sheets	$d_{SCS,Al}$	50 µm [28]
Thickness aluminum double coated	$d_{DCS,Al}$	15 µm [28]
Thickness copper double coated	$d_{DCS,Cu}$	10 µm [28]
Thickness pouch foil	$d_{Pouch\ foil}$	346 µm*
Intercalation fraction 100% SOC anode	$z_{1,An}$	0.676 [29]
Intercalation fraction 0% SOC anode	$z_{0,An}$	0.181 [29]
Intercalation fraction 100% SOC cathode	$z_{1,Cat}$	0.442 [29]
Intercalation fraction 0% SOC cathode	$z_{0,Cat}$	0.986 [29]
Maximal concentration AM anode	$c_{max,An}$	20530 mol m^{-3}*
Maximal concentration AM cathode	$c_{max,Cat}$	31827 mol m^{-3}*
Concentration conductive salt	c_{LiPF_6}	1200 mol m^{-3}*
SEI thickness	d_{SEI}	50 nm
Battery heat capacity	$C_{p,cell}$	9.43 J K^{-1}*
Battery mass	m_{cell}	11.12 g*

*Calculated details see our previous publication[1].

6 Kinetic parameters

Table 6: Kinetic parameters $k_{0,j}$, and $E_{A,j}$ for all Li ion battery degradation reactions obtained by parameterizing the model with the experiments of Stich et al[5] and Maleki et al.[14]

Reaction	Frequency factor, $k_{0,j}$ mol/s	Activation energy, $E_{A,j}$ kJ/mol
$LiPF_6 \rightleftharpoons LiF + \mathbf{PF_5}$	18	53
$PF_5 + H_2O \rightleftharpoons 2\mathbf{HF} + \mathbf{POF_3}$	35	35
$POF_3 + H_2O \rightarrow \mathbf{HF} + HPO_2F_2$	27.5	37.5
$2LiC_6 + 2C_3H_4O_3(EC) \rightarrow \mathbf{(CH_2OCO_2Li)_2} + C_2H_4 + 2C_6$	$3.2 \cdot 10^{-11*}$	42
$2LiC_6 + C_3H_4O_3\,(EC) \rightarrow \mathbf{Li_2CO_3} + C_2H_4 + 2C_6$	$3.2 \cdot 10^{-11*}$	42
$LiC_6 + H_2O \rightarrow \mathbf{LiOH} + 0.5H_2 + C_6$	$3.2 \cdot 10^{-11*}$	42
$(CH_2OCO_2Li)_2 \rightarrow \mathbf{Li_2CO_3} + C_2H_4 + CO_2 + 0.5\mathbf{O_2}$	$1 \cdot 10^{14}$	148
$Li_2CO_3 + 2HF \rightarrow 2LiF + \mathbf{H_2O} + CO_2$	$8 \cdot 10^{14}$	65
$LiC_6 + LiOH \rightarrow Li_2O + 0.5H_2 + C_6$	350	126
$Li_zCoO_2 \rightarrow zLiCoO_2 + \frac{1-z}{3}\mathbf{O_2} + \frac{1-z}{3}Co_3O_4$	10^{47**}	480^{**}
$3.5O_2 + C_4H_8O_3\,(EMC) \rightarrow 4CO_2 + 4\mathbf{H_2O}$	$2.5 \cdot 10^{53**}$	470^{**}
$2.5O_2 + C_3H_4O_3(EC) \rightarrow 3CO_2 + 2\mathbf{H_2O}$	$2.5 \cdot 10^{53**}$	470^{**}

* mol/m/s, ** Only with such high values in activation energy and frequency factor could the reaction behaviour be reproduced with power law kinetics.

7 Additional figures

Figure 4 shows the parameterization of the reference case against the experimental data of Maleki et al.[14] Figure 5 a) shows the produced heats of the formation reactions of LEDC, Li_2CO_3 and LiOH. Note that LiOH does not occur to a notably extent since the water concentration is too low and most of it has been consumed before substantial

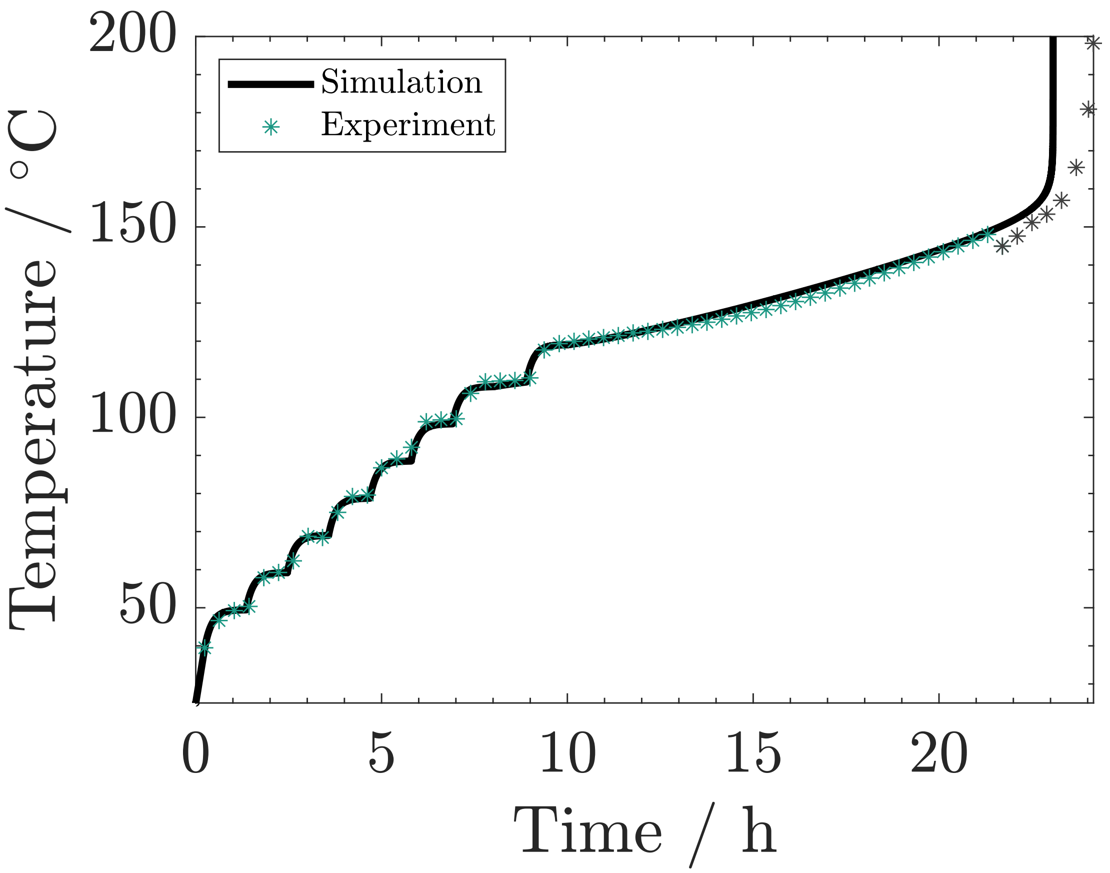

Figure 4: Experimental data from Maleki et al.[13] used for the parameterization of the model. The solid black line indicates the simulation based on the reference scenario. The green and grey stars represent experimental data before and after cell opening, respectively.

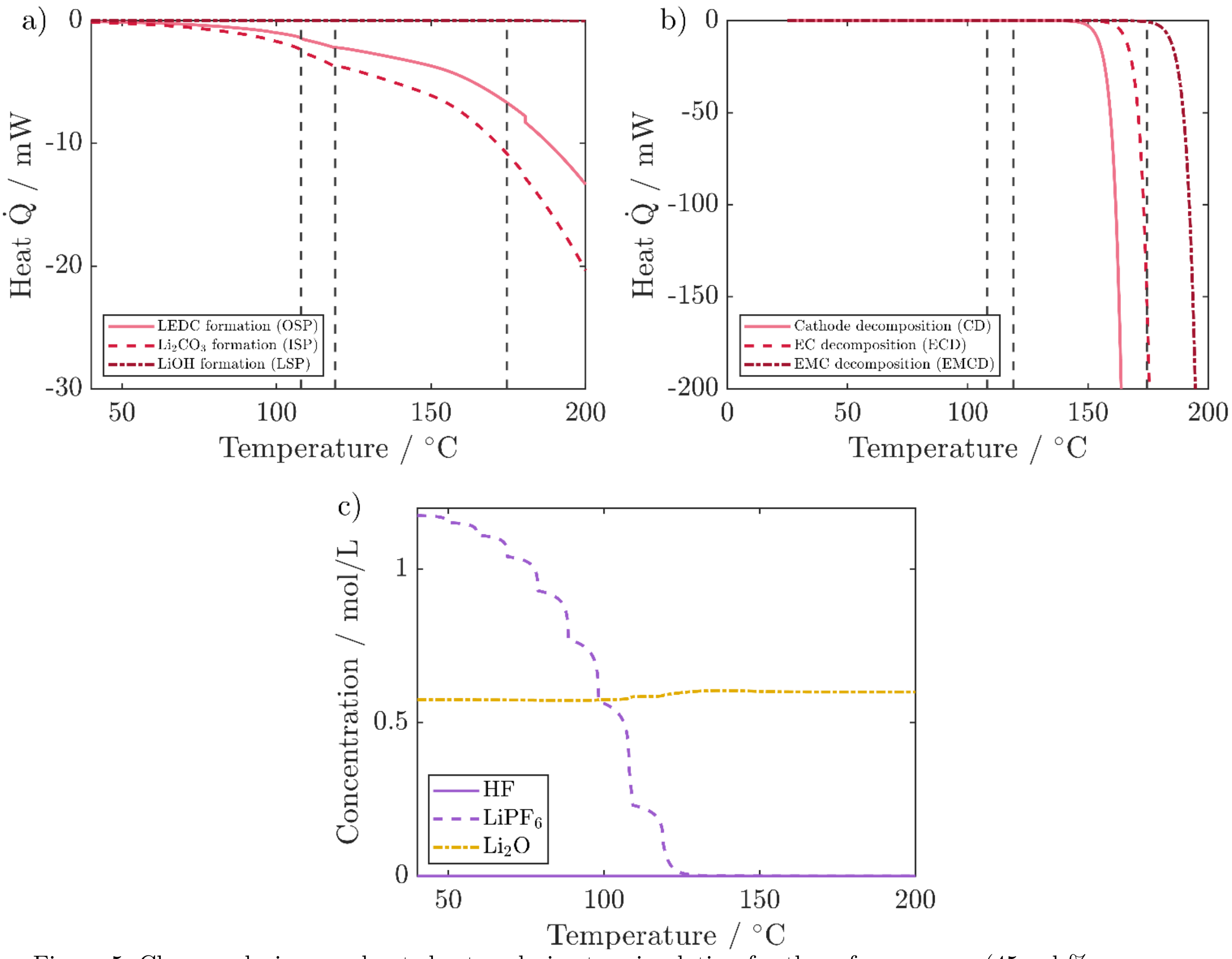

Figure 5: Changes during accelerated rate calorimetry simulation for the reference case (45 vol-% LEDC, 260 ppm H_2O, 50 nm thick SEI): produced heats from a) SEI forming reaction: LEDC, Li_2CO_3, and LiOH, and from b) cathode decomposition, EC decomposition, and EMC decomposition. c) additional concentrations of HF $LiPF_6$ and Li_2O.

formation could take place. Figure 5 b shows the produced heats of cathode, EC and EMC decomposition. Figure 5 c shows the concentrations of HF, $LiPF_6$ and Li_2O.

Figure 6, Figure 7, and Figure 8 show the comprehensive comparison for the SEI composition variation, the SEI thickness variation and the water impurity variation, respectively.

Figure 9 shows the simulation of the variation for water impurities with the alternative temperature step of 2.5 °C compared to the 10 °C of the experiment utilized for model parameterization.

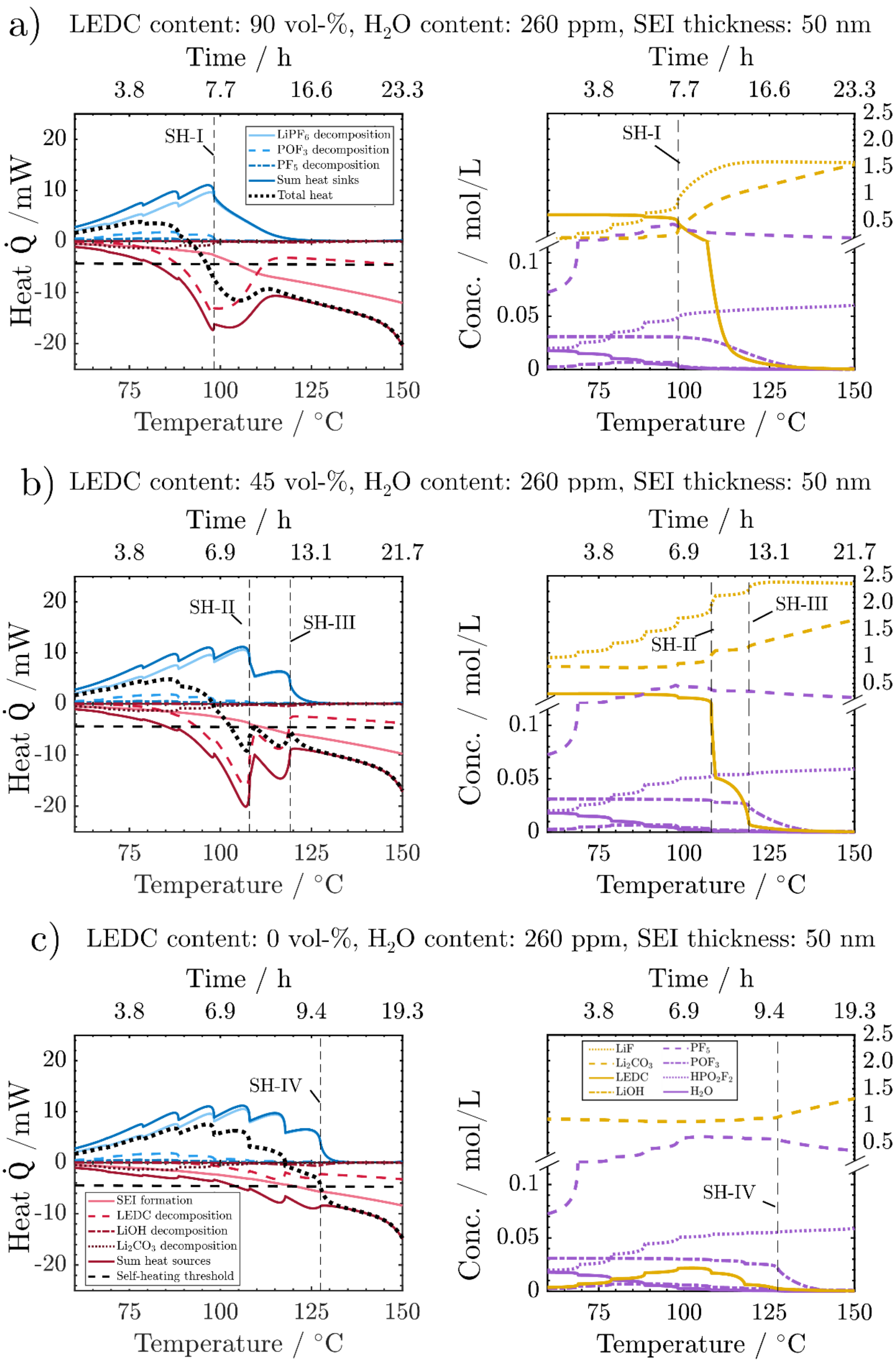

Figure 6: Comprehensive comparison of progression of heat sinks and sources and concentration for the high LEDC (a), the reference (b), and the low LEDC case (c).

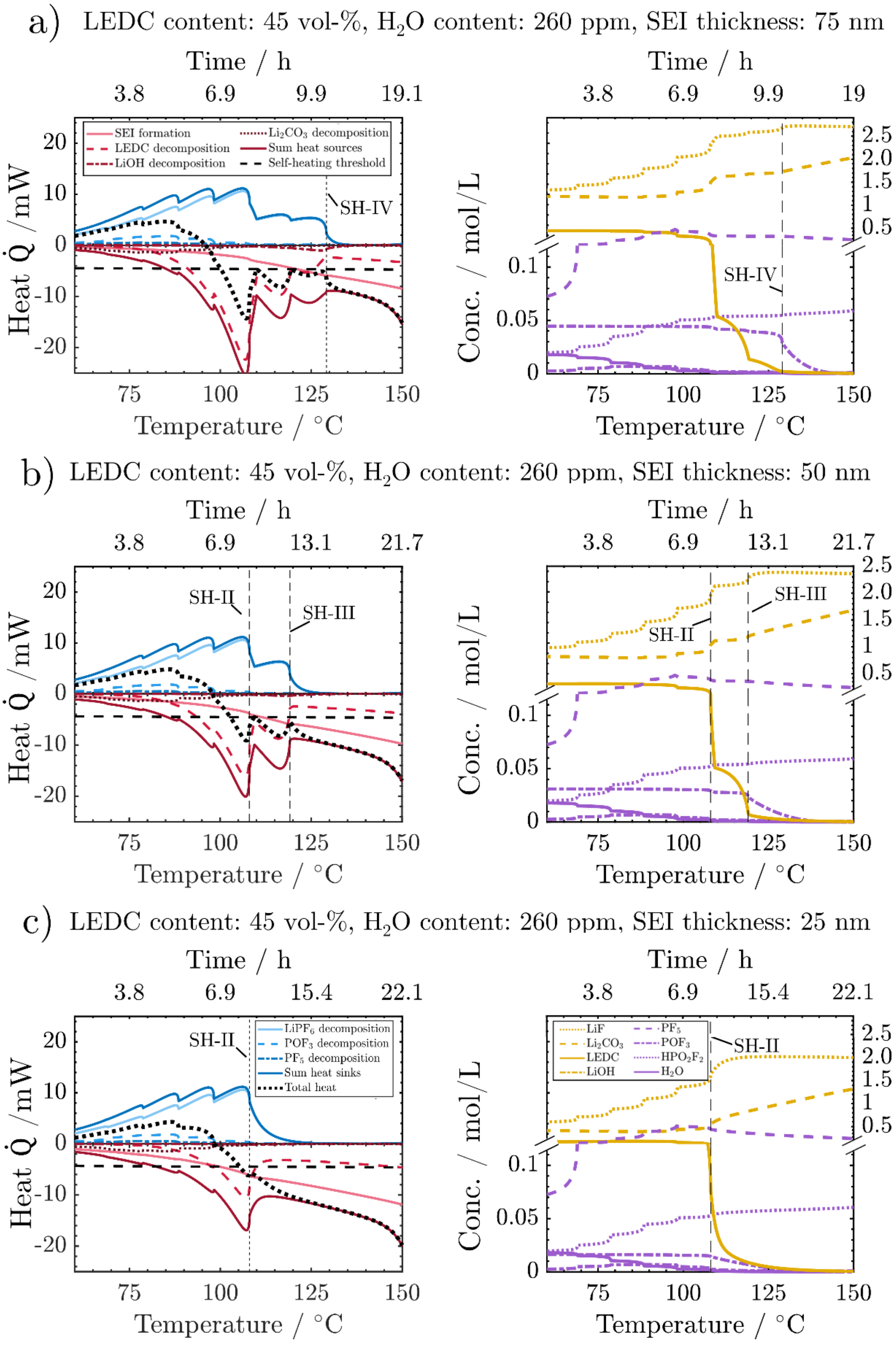

Figure 7: Comprehensive comparison of progression of heat sinks and sources and concentration for the thick SEI (a), the reference (b), and the thin SEI case (c).

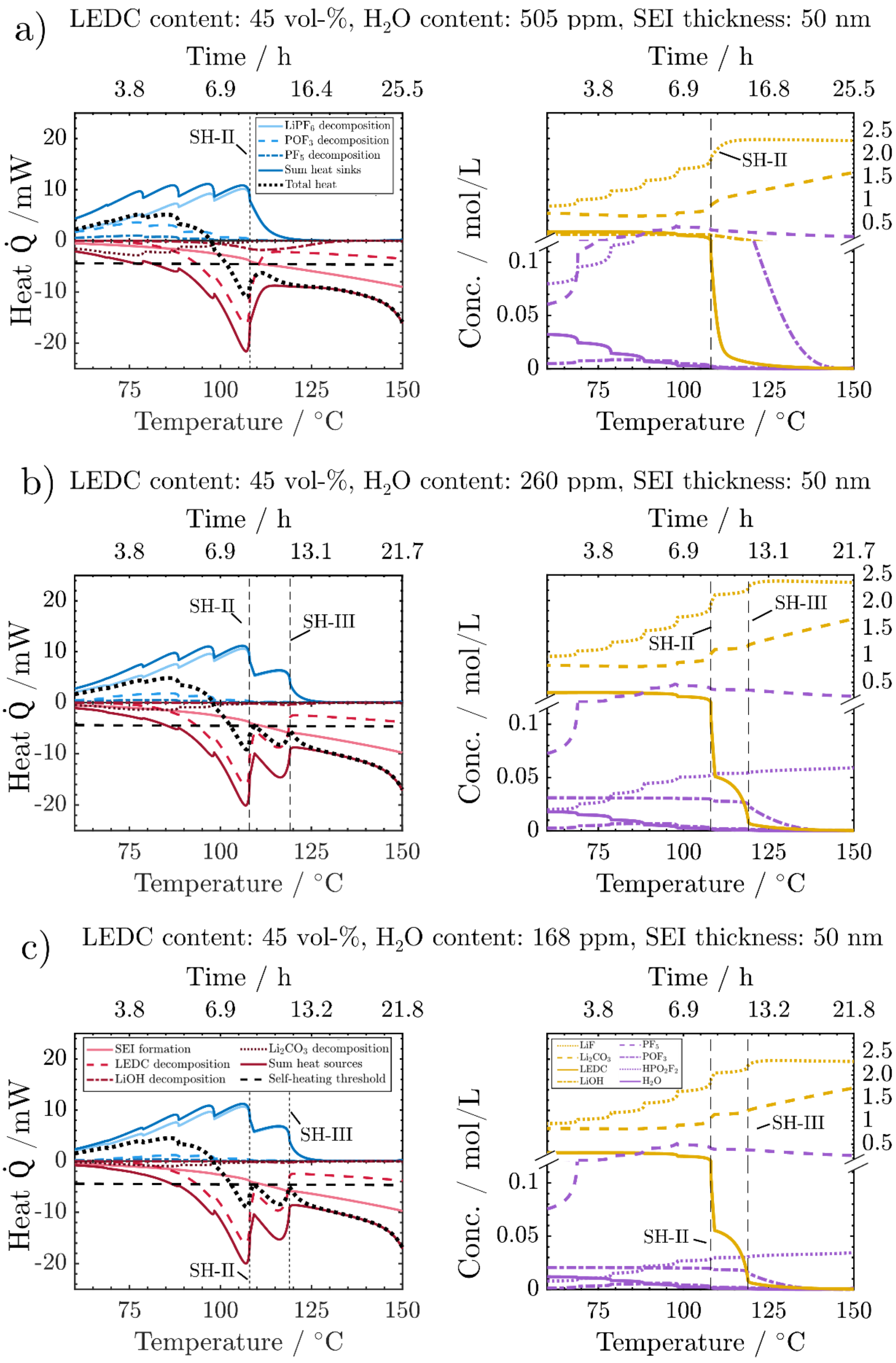

Figure 8: Comprehensive comparison of progression of heat sinks and sources and concentration for the high water contamination (a), the reference (b), and the low water contamination case (c).

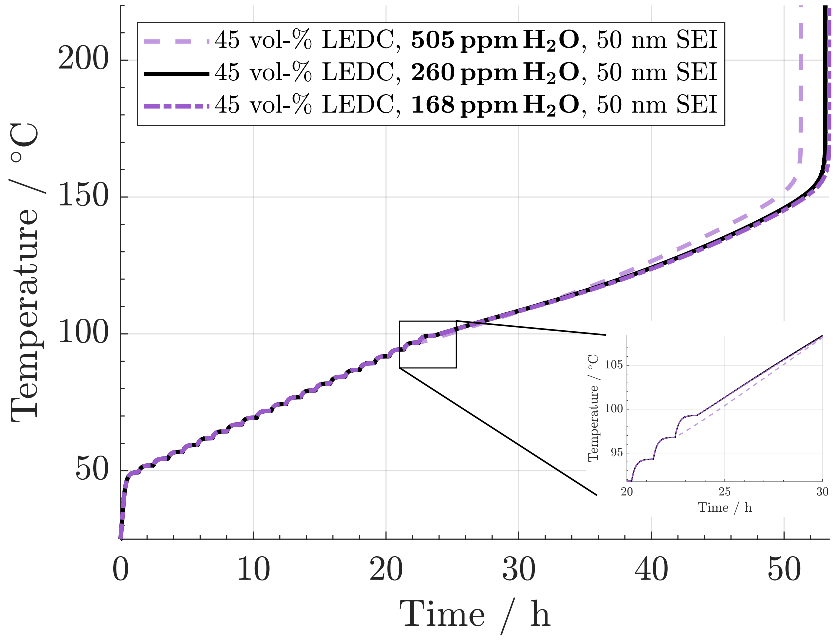

Figure 9: Simulation of the H_2O impurity variation ARC measurements with a 2.5 °C temperature step.

References

[1] F. Baakes, M. Lüthe, M. Gerasimov, V. Laue, F. Röder, P. B. Balbuena, U. Krewer, Journal of Power Sources 2022, 522, 230881.

[2] Sigma Aldrich, Product specification: Lithium hexafluorophosphate solution in ethlene carbonate and dimethyl carbonate 2022, https://www.sigmaaldrich.com/specification-sheets/401/279/746711-BULK_______ALDRICH__.pdf.

[3] F. Huttner, W. Haselrieder, A. Kwade, Energy Technol. 2020, 8, 1900245.

[4] T. Liu, L. Lin, X. Bi, L. Tian, K. Yang, J. Liu, M. Li, Z. Chen, J. Lu, K. Amine, K. Xu, F. Pan, Nature nanotechnology 2019, 14, 50.

[5] M. Stich, M. Göttlinger, M. Kurniawan, U. Schmidt, A. Bund, J. Phys. Chem. C 2018, 122, 8836.

[6] J. W. Abbott, F. Hanke, Journal of chemical theory and computation 2022, 18, 925.

[7] K. Kwon, F. Kong, F. McLarnon, J. W. Evans, J. Electrochem. Soc. 2003, 150, A229.

[8] J. E. HURST, B. KEITH HARRISON, Chemical Engineering Communications 1992, 112, 21.

[9] C. Kupper, W. G. Bessler, J. Electrochem. Soc. 2017, 164, A304-A320.

[10] P. Linstrom, NIST Chemistry WebBook, NIST Standard Reference Database 69, National Institute of Standards and Technology 1997, http://webbook.nist.gov/chemistry/.

[11] Institut f¨ur Arbeitsschutz der Deutschen Gesetzlichen Unfallversicherung, GESTIS - Stoffdatenbank 2020.

[12] M. W. Chase, NIST-JANAF thermochemical tables 4, American Institute of Physics, Woodbury, N.Y. 1998.
[13] K. S. Gavrichev, G. A. Shartpataya, L. N. Golushina, V. N. Plakhotnik, I. V. Goncharova, Russian Journal of Inorganic Chemistry 2002.
[14] H. Maleki, G. Deng, A. Anani, J. Howard, J. Electrochem. Soc. 1999, 146, 3224.
[15] V. V. Avdeev, A. P. Savchenkova, L. A. Monyakina, I. V. Nikol'skaya, A. V. Khvostov, Journal of Physics and Chemistry of Solids 1996, 57, 947.
[16] S. O. Dang, Modelling thermodynamic properties of intercalation compounds for Lithium ion batteries, Forschungszentrum Jülich GmbH, Jülich 2015.
[17] P. Gotcu, H. J. Seifert, Physical chemistry chemical physics PCCP 2016, 18, 10550.
[18] M. Wang, Solid State Ionics 2004, 166, 167.
[19] I. H. Bell, J. Wronski, S. Quoilin, V. Lemort, Industrial & Engineering Chemistry Research 2014, 53, 2498.
[20] M. Łencka, A. Anderko, AIChE J. 1993, 39, 533.
[21] D.-Y. Peng, D. B. Robinson, Ind. Eng. Chem. Fund. 1976, 15, 59.
[22] National Center for Biotechnology Information, PubChem Compound Summary for CID 61681, Difluorophosphoric acid, https://pubchem.ncbi.nlm.nih.gov/compound/Difluorophosphoric-acid.
[23] J. W. Larson, Polyhedron 1991, 10, 1695.
[24] C. L. Yaws, Chemical properties handbook, McGraw-Hill, New York [u.a.] 1999.
[25] Y.-J. Fang, J.-M. Qian, J. Chem. Eng. Data 2005, 50, 340.
[26] V. Pokorný, V. Štejfa, M. Fulem, C. Červinka, K. Růžička, J. Chem. Eng. Data 2017, 62, 4174.
[27] Renata batteries datasheet, ICP622540PMT 2019.
[28] B. Rieger, S. Schlueter, S. V. Erhard, J. Schmalz, G. Reinhart, A. Jossen, Journal of Energy Storage 2016, 6, 213.
[29] A. M. Colclasure, R. J. Kee, Electrochimica Acta 2010, 55, 8960.
